# Structural and biochemical mechanisms of NLRP1 inhibition by DPP9

**DOI:** 10.1038/s41586-021-03320-w

**Published:** 2021-03-17

**Authors:** Menghang Huang, Xiaoxiao Zhang, Gee Ann Toh, Qin Gong, Jia Wang, Zhifu Han, Bin Wu, Franklin Zhong, Jijie Chai

**Affiliations:** 1 Beijing Advanced Innovation Center for Structural Biology, Tsinghua-Peking Joint Center for Life Sciences, Center for Plant Biology, School of Life Sciences, Tsinghua University, Beijing, China; 2 Lee Kong Chian School of Medicine, Nanyang Technological University, Singapore, Singapore; 3 School of Biological Sciences, Nanyang Technological University, Singapore, Singapore; 4 Institute of Structural Biology, Nanyang Technological University, Singapore, Singapore; 5 Skin Research Institute of Singapore (SRIS), Singapore, Singapore; 6 Max Planck Institute for Plant Breeding Research, Cologne, Germany; 7 Institute of Biochemistry, University of Cologne, Cologne, Germany

**Keywords:** Cryoelectron microscopy, NOD-like receptors

## Abstract

Nucleotide-binding domain, leucine-rich repeat receptors (NLRs) mediate innate immunity by forming inflammasomes. Activation of the NLR protein NLRP1 requires autocleavage within its function-to-find domain (FIIND)^1–7^. In resting cells, the dipeptidyl peptidases DPP8 and DPP9 interact with the FIIND of NLRP1 and suppress spontaneous NLRP1 activation^8,9^; however, the mechanisms through which this occurs remain unknown. Here we present structural and biochemical evidence that full-length rat NLRP1 (rNLRP1) and rat DPP9 (rDPP9) form a 2:1 complex that contains an autoinhibited rNLRP1 molecule and an active UPA–CARD fragment of rNLRP1. The ZU5 domain is required not only for autoinhibition of rNLRP1 but also for assembly of the 2:1 complex. Formation of the complex prevents UPA-mediated higher-order oligomerization of UPA–CARD fragments and strengthens ZU5-mediated NLRP1 autoinhibition. Structure-guided biochemical and functional assays show that both NLRP1 binding and enzymatic activity are required for DPP9 to suppress NLRP1 in human cells. Together, our data reveal the mechanism of DPP9-mediated inhibition of NLRP1 and shed light on the activation of the NLRP1 inflammasome.

## Abstract (web summary)

The 2:1 complex between the inflammatory mediator NLRP1 and the dipeptidyl peptidase DPP9 functions to sequester an inflammasome-forming fragment of NLRP1 and enhance NLRP1 autoinhibition.

## Main

**Fig. 1:**
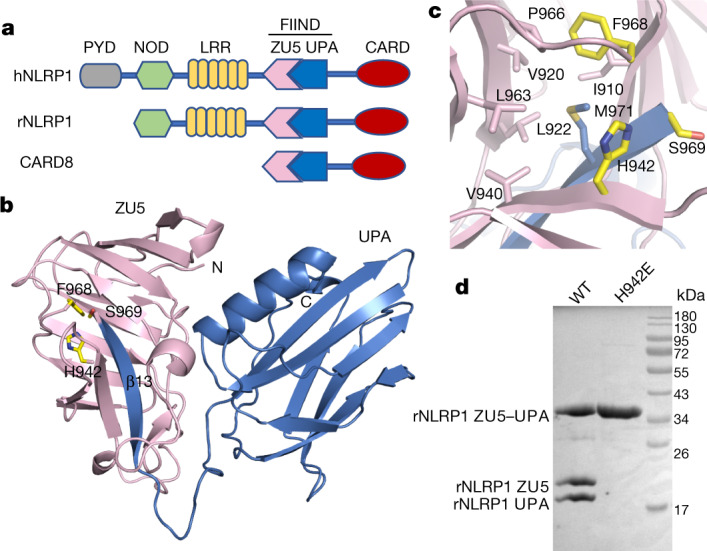
Crystal structure of the FIIND of rNLRP1. **a**, Schematic of domain structures of hNLRP1, rNLRP1 and CARD8. CARD, caspase activation and recruitment domain; PYD, pyrin domain. **b**, Crystal structure of the rNLRP1 FIIND. The ZU5 and UPA subdomains are shown in pink and blue, respectively. The catalytic residues of the FIIND are labelled and shown in stick representation. **c**, A close-up view of the catalytic site of the FIIND. **d**, Mutation of the catalytic residue H942 abolishes autoproteolysis of the rNLRP1 FIIND. Wild-type and H942 mutant rNLRP1 FIIND proteins were purified from insect cells and visualized by SDS–PAGE followed by Coomassie blue staining. See Supplementary Fig. 1 for gel raw data.

In the mammalian innate immune system, the detection of pathogen-derived or host-derived signals by NLRs induces their oligomerization, forming multiprotein complexes called inflammasomes that mediate inflammatory cell death and cytokine secretion^10^. NLRs generally consist of an N-terminal domain, a central nucleotide-binding and oligomerization domain (NOD) and a C-terminal leucine-rich repeat (LRR) domain. NLRP1, like CARD8 (refs. ^4,7^), contains an unusual domain known as FIIND^11^ (Fig. 1a), which contains the subdomains ZU5 (found in the tight-junction protein ZO-1 and the netrin receptor UNC5) and UPA (conserved in UNC5, the death-domain-containing protein PIDD and proteins of the ankyrin family). Autoproteolysis between these two subdomains is a prerequisite for the activation of NLRP1 (refs. ^4,7^) and of CARD8 (ref. ^12^). *Bacillus anthracis* lethal factor is the best-characterized pathogen-derived trigger for the activation of rodent NLRP1 (refs. ^5,13^). Lethal factor cleaves mouse NLRP1B close to its N terminus and induces proteasomal degradation of the entire N-terminal NOD–LRR–ZU5 fragment via the N-end rule pathway^1–3^. This liberates the active UPA–CARD fragment that rapidly oligomerizes to engage downstream inflammasome effectors such as apoptosis-associated speck-like protein containing a CARD (ASC) and pro-caspase-1 (refs. ^1,3^). This unique mechanism involving ‘functional degradation’ is conserved in the activation of human NLRP1 (hNLRP1) by the 3C proteases of enteroviruses^14^ and in the activation of CARD8 by HIV-1 protease^15^.

DPP8 and DPP9 are related intracellular prolyl peptidases that are implicated in immune regulation and in other viral cellular processes^16^. They are endogenous inhibitors of the NLRP1 inflammasome in humans^8,9,12^ and in rodents^6,8,12,17,18^. Notably, the FIIND of hNLRP1 is necessary and sufficient for interaction with human DPP9 (hDPP9) (ref. ^9^). Furthermore, inhibitors of class IV DPPs—such as valine boroproline (VbP)—or knockout of DPP8 and DPP9 specifically activate NLRP1 and/or CARD8 (refs. ^6,8,9,12^). Like lethal factor, VbP also induces the proteasome-mediated degradation of the NLRP1B N-terminal fragment, but this induction is independent of N-degron recognition. VbP and lethal factor therefore trigger rodent NLRP1 activation through two distinct, independent pathways. At present, the mechanisms that underlie the DPP9-mediated inhibition of NLRP1 remain unknown.

## Crystal structure of the autoinhibited FIIND

**Extended Data Fig. 1:**
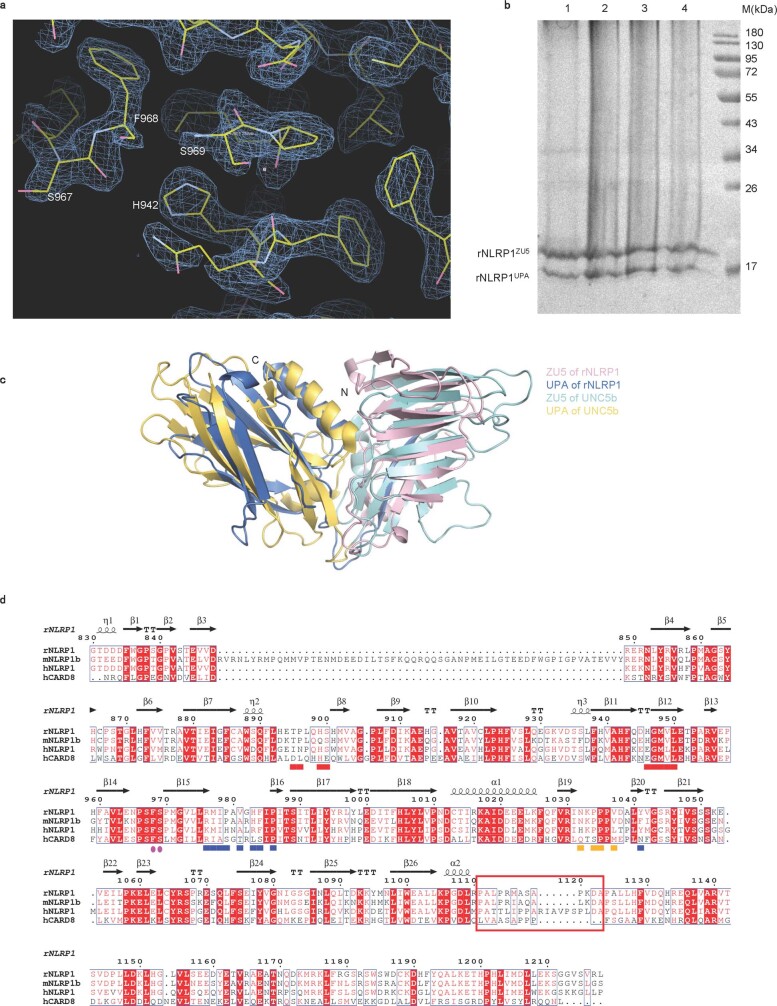
Complete autocleavage of rNLRP1 FIIND in crystals and sequence alignment among rNLRP1, hNLRP1, NLRP1B and CARD8. **a**, Electron density around the active site of rNLRP1 FIIND. The autocleavage site is between F968 and S969 of rNLRP1. **b**, SDS–PAGE analysis of the crystals of rNLRP1 FIIND. The crystals were collected and dissolved in SDS–PAGE buffer. Lanes 1–4 represent crystals from four different wells. **c**, Structural alignment of rNLRP1 FIIND and UNC5b. The crystal structure of rNLRP1 FIIND was aligned with that of UNC5b (PDB code: 3G5B). The colour code is indicated on the figure. **d**, Sequence alignment of the FIIND-CARD domains of rNLRP1, hNLRP1, NLRP1B and CARD8. The rDPP9-interacting residues around the ZU5-binding site, the UPA-binding site and residues at the UPA homodimeric interface are highlighted with red, blue and yellow squares, respectively. The two residues from the catalytic FS motif are indicated by purple dots, and the linker region between UPA and CARD is highlighted within the red box. See Supplementary Fig. 1 for gel raw data.

A crystal structure of the rNLRP1 FIIND of rNLRP1 shows that the purified protein is autocleaved at the predicted position between F968 and S969 (Extended Data Table 1, Extended Data Fig. 1a). This is further confirmed by SDS–PAGE analysis of the crystals (Extended Data Fig. 1b). The structure of rNLRP1 FIIND resembles that of the autoinhibited netrin receptor UNC5b^19^ (Extended Data Fig. 1c). Inter-domain interaction between ZU5 and UPA largely involves the first β-strand (β13) of UPA, which forms two anti-β sheets with ZU5 (Fig. 1b); this explains how the ZU5 domain can block the release of the active UPA–CARD fragment^4,7^.

The catalytically essential FS motif^20^ is conserved in NLRP1 homologues and in CARD8 (Extended Data Fig. 1d). In the structure of rNLRP1, F968 from this motif points into a hydrophobic pocket (Fig. 1c), as is observed for the corresponding phenylalanine residue in the FIIND-containing proteins NUP98 (ref. ^21^) and PIDD^22^. H942 of rNLRP1, which is highly conserved in NLRP1 proteins and in CARD8 (Extended Data Fig. 1d), is located adjacent to S969 from the FS motif (Fig. 1c). Mutation of H942 of rNLRP1 resulted in a complete loss of autocleavage (Fig. 1d), as was previously observed after mutation of the corresponding residues H270 of CARD8 or H1186 of hNLRP1 (ref. ^20^). Collectively, these data suggest that H942 of rNLRP1 is a catalytic residue.

## Architecture of the 2:1 rNLRP1–rDPP9 complex

**Fig. 2:**
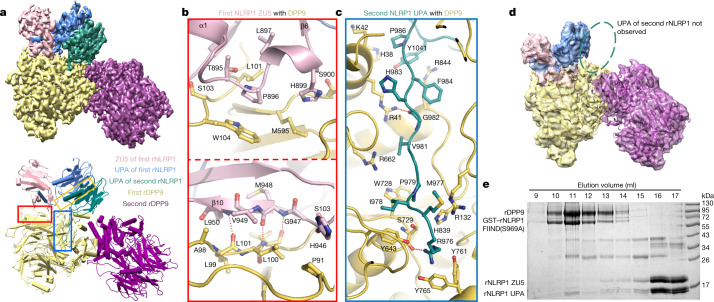
Assembly mechanism of the 2:1 rNLRP1–rDPP9 complex. **a**, Top, the final cryo-EM density of the rNLRP1–rDPP9 complex at 3.18 Å. Colour codes for domain structures are indicated. Bottom, model of the 2:1 rNLRP1–rDPP9 complex. The three interfaces that mediate the rNLRP1–rDPP9 interaction are shown in coloured boxes. Red, ZU5-binding site; blue, UPA-binding site; yellow, UPA–UPA-binding site. **b**, Detailed interactions between ZU5 and rDPP9 at the ZU5-binding site within the red-framed region in **a**. Hydrogen-bonding interactions are indicated by red dashed lines. **c**, Detailed interactions between UPA and rDPP9 at the UPA-binding site within the blue-framed region in **a**. **d**, 3D reconstruction of the rNLRP1 FIIND–CARD(S969A)–rDPP9 complex. Highlighted within the open ellipse is the vacant UPA-binding site. **e**, The ZU5 subdomain is displaced after interaction of the second rNLRP1 with rDPP9. The preformed rNLRP1 FIIND(S969A)–rDPP9 complex was incubated with fully autoprocessed rNLRP1 FIIND and analysed by gel-filtration experiments. Protein fractions from the gel-filtration assay were visualized by SDS–PAGE followed by Coomassie-blue staining. See Supplementary Fig. 1 for gel raw data.

**Extended Data Fig. 2:**
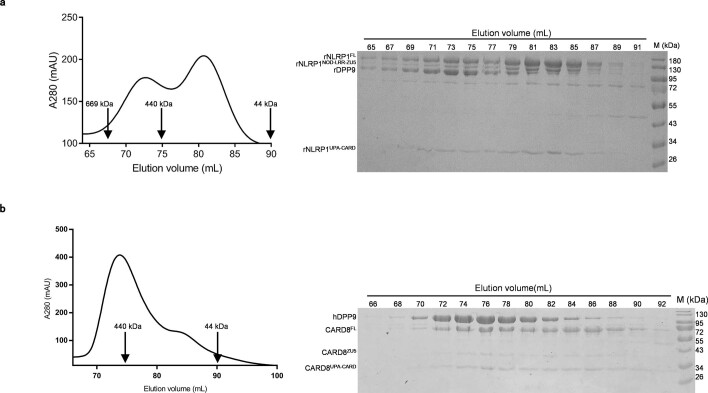
Gel-filtration analysis of the rNLRP1–rDPP9 and the CARD8–hDPP9 interactions. **a**, Full-length GST–rNLRP1 and rDPP9 were expressed in insect cells. The complex was purified through GS4B resin. After elution, GST was removed using precision protease and the complex was subjected to gel filtration. Left, the gel-filtration profile of the complex. Right, the protein fractions were visualized by SDS–PAGE followed by Coomassie-blue staining. **b**, The interaction between full-length GST–CARD8 and hDPP9 was assayed as described in **a**. See Supplementary Fig. 1 for gel raw data.

**Extended Data Fig. 3:**
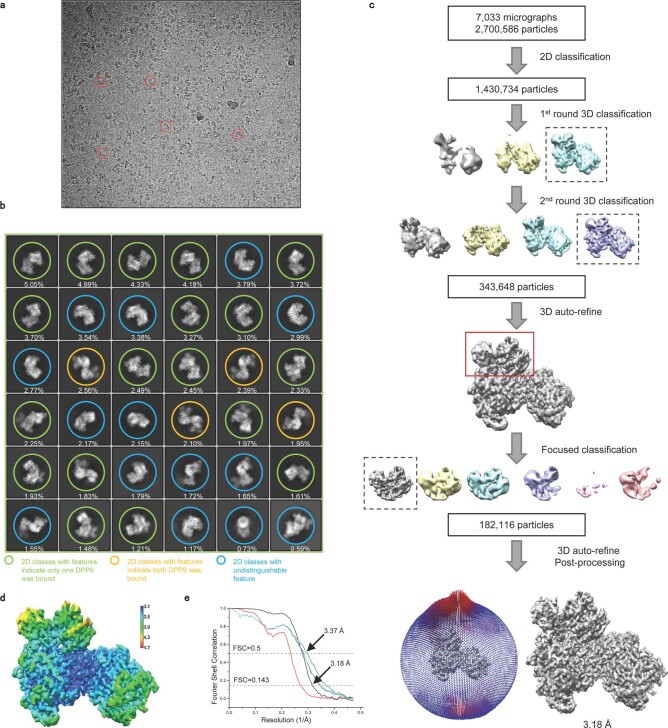
3D reconstruction of the rNLRP1–rDPP9 complex. **a**, A representative cryo-EM image of the rNLRP1–rDPP9 complex. **b**, Representative views of 2D class averages of the rNLRP1–rDPP9 complex used for 3D reconstruction. 2D classes with different rDPP9-binding features are shown in different colours. **c**, Flow chart of cryo-EM data processing and 3D reconstruction of the rNLRP1–rDPP9 complex. **d**, The final EM density map of the rNLRP1–rDPP9 complex. The colour code shows the local resolution estimated using Relion. **e**, Fourier shell correlation curves at 0.143 for the final model of the rNLRP1–rDPP9 complex unmasked (red) or masked (black), and at 0.5 for rNLRP1–rDPP9 global model refined against the 3.18 Å rNLRP1–rDPP9 global map (green), the first half map (blue) and the second half map (cyan). The small difference between the blue and cyan curves after the estimated resolution indicates no overfitting of the coordinate refinement.

Gel-filtration experiments confirmed the formation of a stable complex between full-length rNLRP1 and rDPP9 proteins purified from insect cells (Extended Data Fig. 2a)—consistent with the results of previous studies^3,8,9^. A similar result was also obtained using hDPP9 and CARD8 (Extended Data Fig. 2b). After purification by gel filtration, the rNLRP1–rDPP9 complex was analysed by cryo-electron microscopy (cryo-EM) (Extended Data Fig. 3). Two-dimensional class averages showed that rDPP9 formed dimers, but only one subunit was bound to NLRP1 in most of the particles (Extended Data Fig. 3b). We used the particles with one rDPP9 subunit bound by rNLRP1 for further cryo-EM analysis. After 3D classification, a subset of 182,116 particles was used for final 3D reconstruction, generating a map with a global resolution of 3.18 Å (Fig. 2a, Extended Data Fig. 3c–e, Extended Data Table 2).

**Extended Data Fig. 4:**
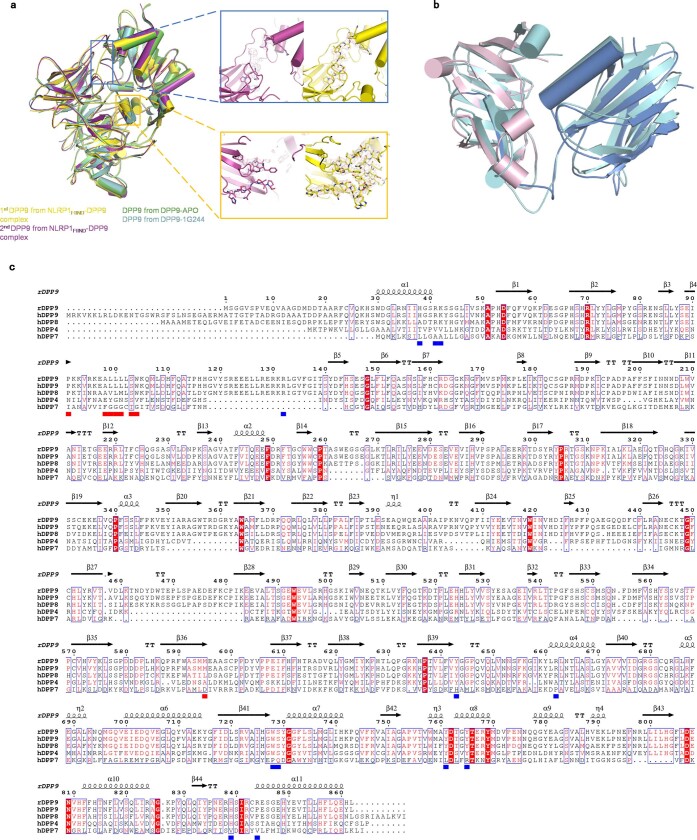
Structural and sequence alignment. **a**, Left, structural alignment between the first rDPP9, second rDPP9, apo-hDPP9 and hDPP9–1G244 (in cartoon representation). The colour code is indicated on the figure. Better-defined loop regions in the rNLRP1-bound rDPP9 subunit are highlighted with open frames. Right, electron density around the stabilized loop regions induced by rNLRP1. All densities are generated in PyMOL and contoured at 5 (region coloured blue) or 7 (region coloured yellow) sigma. **b**, Alignment of the cryo-EM structure of the rNLRP1 FIIND (with ZU5 shown in pink and UPA in blue) with the crystal structure of the rNLRP1 FIIND (in cyan). **c**, Sequence alignment of rDPP9, hDPP9, hDPP8, hDPP4 and hDPP7. The rNLRP1-interacting residues from the ZU5- and UPA-binding site are marked with red and blue squares, respectively.

rDPP9 forms a homodimer (Fig. 2a) that is nearly identical to the dimeric hDPP9 (ref. ^23^). Several flexible loop regions were not observed in the reported apo-hDPP9 structure, but their equivalents are well defined in the structure of the rNLRP1-bound rDPP9 (Extended Data Fig. 4a). Unexpectedly, the liganded rDPP9 subunit is bound by two rNLRP1 molecules (Fig. 2a), which we hereafter term the 2:1 rNLRP1–rDPP9 complex. The first rNLRP1 molecule contains the complete FIIND, which has a conformation nearly identical to that observed in the crystal structure of the free FIIND (Extended Data Fig. 4b); the remaining domains of this rNLRP1 molecule are not discernible in the cryo-EM density. In the second rNLRP1 molecule, only the UPA domain is well defined.

Three surfaces mediate the formation of the 2:1 rNLRP1–rDPP9 complex (Fig. 2a). The first surface is mediated by ZU5 of rNLRP1, which binds to one lateral side of the β-propeller domain of rDPP9 (termed the ZU5-binding site of rDPP9) (Fig. 2a, b, red box). The second interface is formed by the deep insertion of an N-terminal loop (N-loop) from the UPA domain of the second rNLRP1 molecule into the rDPP9 substrate-binding channel (termed the UPA-binding site) (Fig. 2a, c, blue box). Notably, the UPA N-loop forms a β-sheet with ZU5 in the autoinhibited FIIND (Fig. 1b). Additionally, the homodimerization interface of the two UPA domains also contributes to formation of the 2:1 complex (termed the UPA dimerization site) (Fig. 2a, yellow box).

## Specific interaction between rNLRP1 and rDPP9

**Extended Data Fig. 5:**
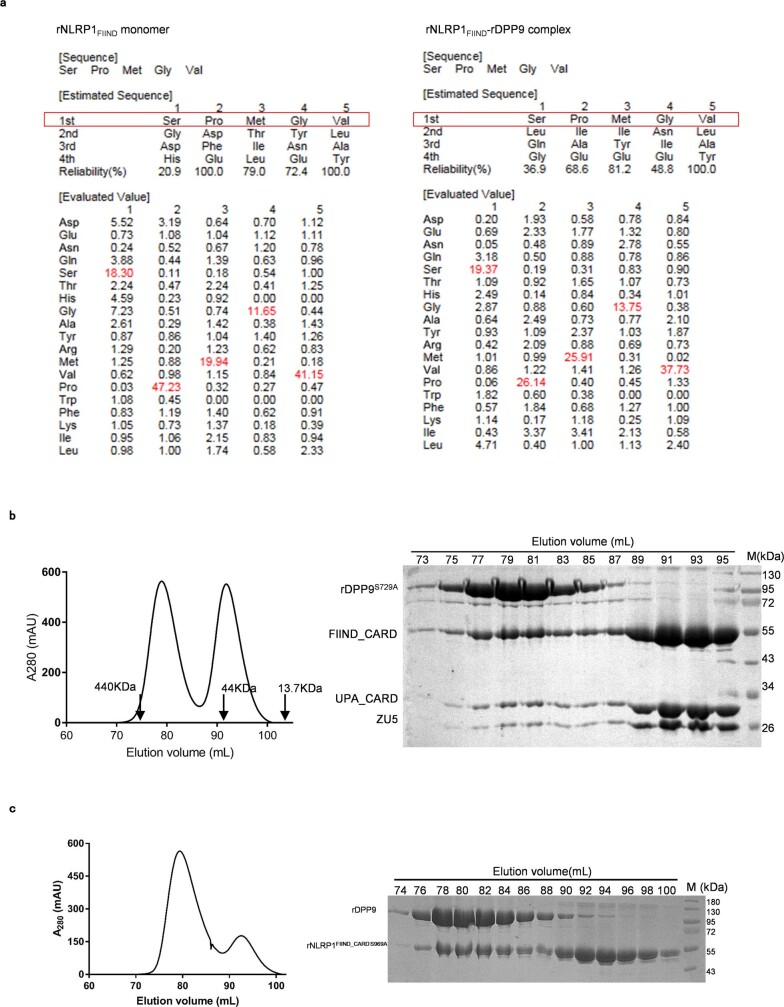
Functional assays to analyse the influence of DPP9 protease activity on the interaction between DPP9 and NLRP1. **a**, N-terminal sequence analysis of rNLRP1 FIIND by Edman degradation. Shown on the left and the right are N-terminal sequences of UPA from rNLRP1 FIIND and rNLRP1 FIIND–rDPP9, respectively. **b**, Catalytic activity of rDPP9 is dispensable for the binding of rDPP9 to rNLRP1. The catalytic mutant rDPP9(S729A) with GST fused at the N terminus was co-expressed with rNLRP1 FIIND–CARD in insect cells. The complex was purified through GS4B resin. After elution, GST was removed using precision protease and the complex was subjected to gel filtration. Left, the gel-filtration profile of the complex. Right, the protein fractions were visualized by SDS–PAGE followed by Coomassie-blue staining. **c**, Gel-filtration analysis of the autoprocessing-deficient S969A mutant of rNLRP1 FIIND-CARD in complex with rDPP9. The two proteins were co-expressed in insect cells and purified using affinity chromatography. The purified complex was further cleaned using gel filtration. Left, the gel-filtration profile of the complex. Right, the fractions of the two proteins were visualized by SDS–PAGE followed by Coomassie-blue staining. See Supplementary Fig. 1 for gel raw data.

The interactions within the ZU5-interacting site consist of extensive contacts between β10, the α1–β6 loop of the rNLRP1 FIIND ZU5 subdomain, and the β-propeller domain of rDPP9 (Fig. 2b). β10 of FIIND forms three main-chain hydrogen bonds with the N-terminal portion of a long loop of rDPP9, forming an anti-β-sheet-like structure (Fig. 2b). The rDPP9 loop also makes hydrophobic contacts with the middle part of the α1–β6 loop of FIIND. The amino acids of this ZU5-interacting site are conserved in DPP8 but not in DPP4 (Extended Data Fig. 4c), which explains why DPP4 fails to inhibit NLRP1B^8,24^. In addition, the short α-helix in the α1–β6 loop of FIIND packs against the other two loops of rDPP9. The substrate-binding groove of rDPP9 is completely blocked by the UPA N-loop (Fig. 2c). Consistent with previous studies^8,9,25^, N-terminal sequencing indicated that the seven non-structured, N-terminal residues of the UPA N-loop were not cleaved by DPP9 (Extended Data Fig. 5a).

## rNLRP1(S969A) forms a 1:1 complex with rDPP9

**Extended Data Fig. 6:**
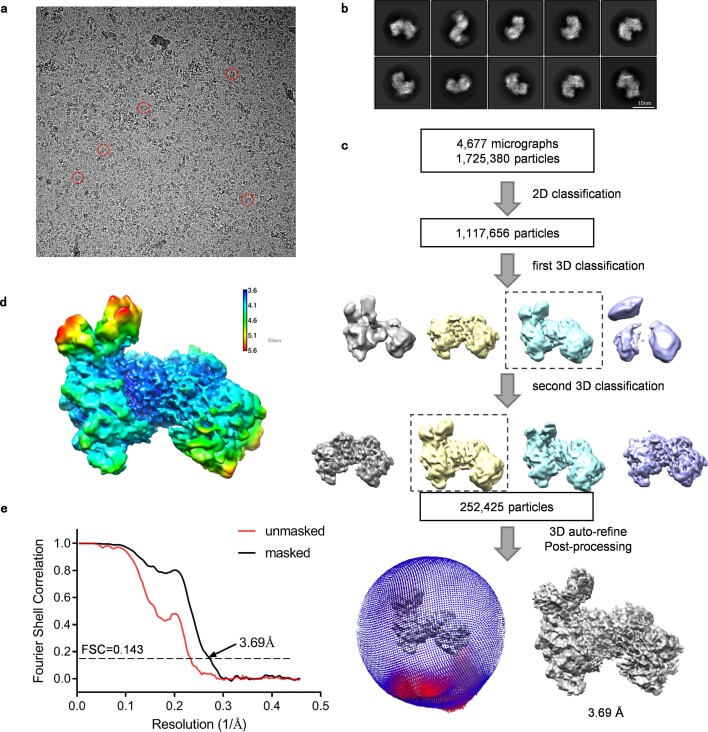
3D reconstruction of the rNLRP1 FIIND-CARD(S969A)–rDPP9 complex. **a**, A representative cryo-EM image of the rNLRP1 FIIND–CARD(S969A)–rDPP9 complex. **b**, Representative 2D class averages of the rNLRP1 FIIND–CARD(S969A)–rDPP9 complex. **c**, Flow chart of cryo-EM data processing and 3D reconstruction of the rNLRP1 FIIND–CARD(S969A)–rDPP9 complex. **d**, The final EM density map of the rNLRP1 FIIND–CARD(S969A)–rDPP9 complex. The colour code shows the local resolution estimated using Relion. **e**, Fourier shell correlation curve (at 0.143) of the final reconstruction of the rNLRP1 FIIND–CARD(S969A)–rDPP9 complex.

The catalytically inactive mutant rDPP9(S729A)—in which the serine residue at position 729 is mutated to alanine—formed a stable complex with the rNLRP1 FIIND–CARD fragment, as determined by gel-filtration experiments (Extended Data Fig. 5b). rNLRP1 FIIND in the first bound position adopts a nearly identical conformation to that of free rNLRP1 FIIND in the crystal structure (Extended Data Fig. 4b), which suggests that a non-autocleavable rNLRP1 can interact with rDPP9. Indeed, the auto-cleavage mutant fragment rNLRP1 FIIND–CARD(S969A) retained rDPP9-binding activity (Extended Data Fig. 5c), consistent with previous observations^8,12^. Notably, the cryo-EM structure of rDPP9 in complex with rNLRP1 FIIND–CARD(S969A) (Extended Data Fig. 6) is nearly identical to that of the wild-type complex encompassing the first rNLRP1 molecule, except that no clear density was found in the second rNLRP1-binding position (Fig. 2d). This result indicates that rNLRP1 FIIND–CARD(S969A) can interact with the ZU5-binding site but not with the UPA-binding site, establishing that autoproteolysis is required for rNLRP1 to fit into the second position to form the 2:1 complex. This could also explain why autoproteolysis-deficient NLRP1 mutants retain DPP9-binding activity, but to a lesser extent^8,9^.

The ZU5 domain of rNLRP1 was not observed in the second position (Fig. 2a), because when rNLRP1 docks into this position this domain becomes either flexible or dissociated. To differentiate between these two possibilities, we tested whether an autocleaved rNLRP1 FIIND fits into the second position in a preformed 1:1 rNLRP1 FIIND(S969A)–rDPP9 complex. Gel filtration showed that the autocleaved FIIND did interact with rNLRP1 FIIND(S969A)–rDPP9 (Fig. 2e). In the resulting complex, the ZU5 and UPA subdomains of rNLRP1 became sub-stoichiometric, which indicates that ZU5 dissociates from UPA after rNLRP1 binding in the second position.

## NLRP1 blocking by DPP9 enzyme and binding activity

**Fig. 3:**
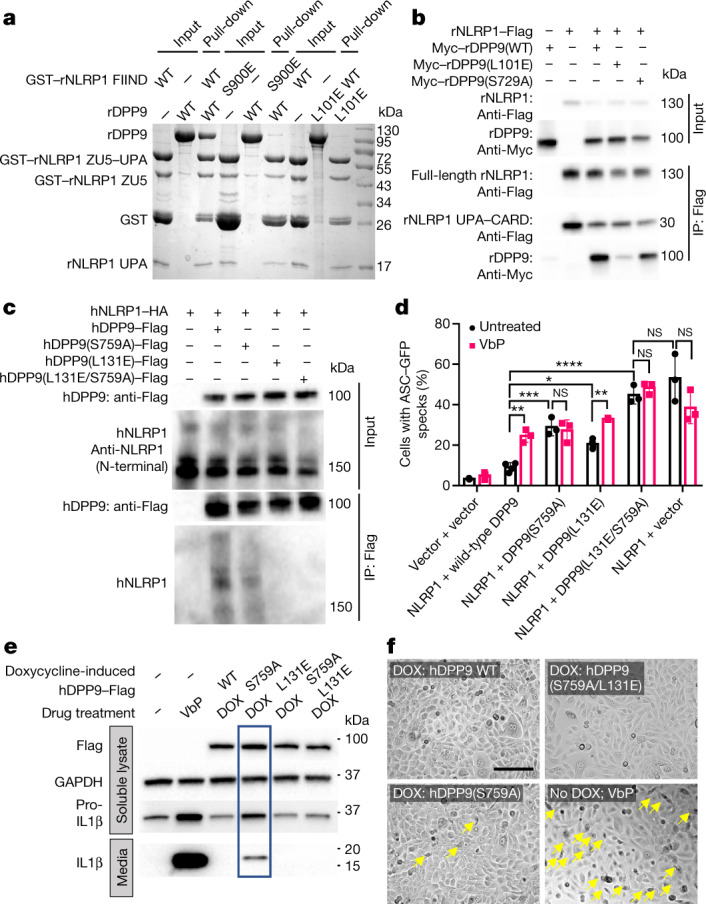
Both protease activity and FIIND binding are important for the inhibition of NLRP1 by DPP9. **a**, rNLRP1 FIIND with an N-terminal GST tag (GST–NLRP1 FIIND) was used to pull down non-tagged rDPP9 in vitro. **b**, Lysates from 293T cells transfected with the indicated Flag-tagged rNLRP1 and Myc-tagged rDPP9 proteins were subjected to anti-Flag immunoprecipitation (IP). **c**, As in **b** using Flag–hDPP9 and haemagglutinin (HA)-tagged hNLRP1. **d**, ASC–GFP DPP8/DPP9 double-knockout 293T cells were transfected with the indicated constructs and treated with and without VbP for 24 h. Bar graphs represent data from biological triplicates. Two-way ANOVA, *P < 0.05, **P < 0.01, ***P < 0.001, ****P < 0.0001, NS, not significant. **e**, Immortalized N/TERT-1 keratinocytes stably transduced with Tet-ON-3×Flag DPP9 lentiviruses were treated with either doxycycline (DOX) or VbP for 24 h. Lysates and the culture media were analysed by immunoblotting. **f**, Images of N/TERT-1 keratinocytes treated as in **e**. Pyroptotic cells are indicated by yellow arrows. Scale bar, 150 μm. See Supplementary Figs. 1 and 3 for gel raw data. Source data

**Extended Data Fig. 7:**
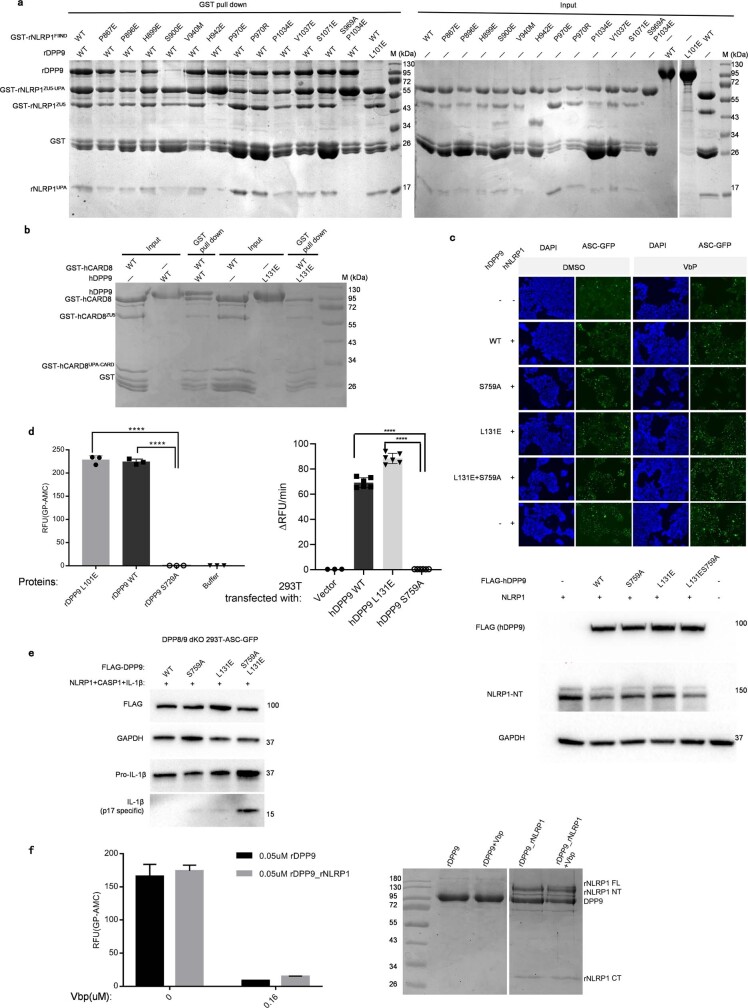
DPP9 interacts with FIIND from rNLRP1 and CARD8. **a**, Mutagenesis analysis of the ZU5-binding site of rDPP9. N-terminally GST-fused wild-type or mutant rNLRP1 FIIND and rDPP9 were individually purified from insect cells. Wild-type or mutant rNLRP1 FIIND was used to pull down rDPP9 (including rDPP9(L101E)) with GS4B resin. After extensive washing, proteins bound to the GS4B resin were visualized by SDS–PAGE followed by Coomassie-blue staining. **b**, The interaction of hDPP9 with CARD8. N-terminally GST-fused CARD8 was used to pull down non-tagged wild-type or mutant hDPP9 in vitro. **c**, Top, representative images of DPP8/DPP9 double-knockout ASC–GFP 293T cells transfected with hNLRP1 and wild-type hDPP9 or hDPP9 mutants. Bottom, expression of wild-type and DPP9 mutants in DPP8/DPP9 double-knockout 293T cells. The soluble lysate was blotted for DPP9, NLRP1 or GAPDH. **d**, Mutation of L101 of rDPP9 (left) or its equivalent L131 of hDPP9 (right) has no effect on the protease activity. Each experiment was repeated at least three times. One-way ANOVA, ****P < 0.0001. RFU, relative fluorescence units. **e**, DPP8/DPP9 double-knockout 293T cells were co-transfected with 3×Flag hDPP9 variant together with CASP1-Myc and pro-IL-1β. ASC–GFP speck formation was monitored for consistency with that in **c**. Lysates were analysed by immunoblotting. **f**, Left, inhibition of the protease activity of rDPP9 and rNLRP1–rDPP9 by VbP. Right, SDS–PAGE analyses of the rDPP9 and rNLRP1–rDPP9 complexes used in the activity assays. See Supplementary Figs. 2 and 4 for gel raw data. Source data

Point mutations at S900 of rNLRP1 and L101 of rDPP9, which were predicted to disrupt the ZU5-binding site, were found to abrogate or markedly compromise the interaction between rNLRP1 and rDPP9 (Fig. 3a, b, Extended Data Fig. 7a). This suggests that the first binding position is required in order for the second rNLRP1 to bind rDPP9. Similarly, mutation of L131 of hDPP9—the residue corresponding to L101 of rDPP9—resulted in no interaction with hNLRP1 at all in 293T cells (Fig. 3c). Notably, the corresponding mutation (L131E) in hDPP9 also resulted in a loss of interaction with CARD8 (Extended Data Fig. 7b). This suggests that the mechanism of ZU5-mediated interaction with DPP9 might be conserved among rNLRP1, hNLRP1 and CARD8.

To determine whether the binding of NLRP1 is functionally important for its inhibition by DPP9, we imaged hDPP8/hDPP9 double-knockout 293T cells that expressed ASC–GFP and co-expressed hNLRP1 and hDPP9 variants (ref. ^9^). As anticipated, wild-type hDPP9 efficiently inhibited the hNLRP1-dependent formation of ASC–GFP specks (Fig. 3d, Extended Data Fig. 7c). By contrast, the binding-deficient but enzymatically active mutant hDPP9(L131E) (Extended Data Fig. 7d) and the catalytically dead but binding-active mutant hDPP9(S759A) were both defective in doing so (Fig. 3d, Extended Data Fig. 7c). These findings indicate that both catalytic activity and binding to the FIIND are important for the hDPP9-mediated inhibition of hNLRP1. hDPP9(L131E)—but not hDPP9(S759A)—retained sensitivity towards VbP (Fig. 3d), which provides further evidence that the protease activity of hDPP9 has a critical role in hNLRP1 inhibition. Furthermore, simultaneous mutations of these two residues were additive in eliminating the inhibitory effect of hDPP9 on the formation of ASC specks (Fig. 3d). Expression of wild-type hDPP9—but not hDPP9(L131E), hDPP9(S759A) or hDPP9(L131E/S759A)—in hDPP8/hDPP9 double-knockout 293T cells fully inhibited IL-1β cleavage by caspase-1 (Extended Data Fig. 7e), further confirming that both catalytic activity and FIIND binding are essential for hNLRP1 inhibition.

**Extended Data Fig. 8:**
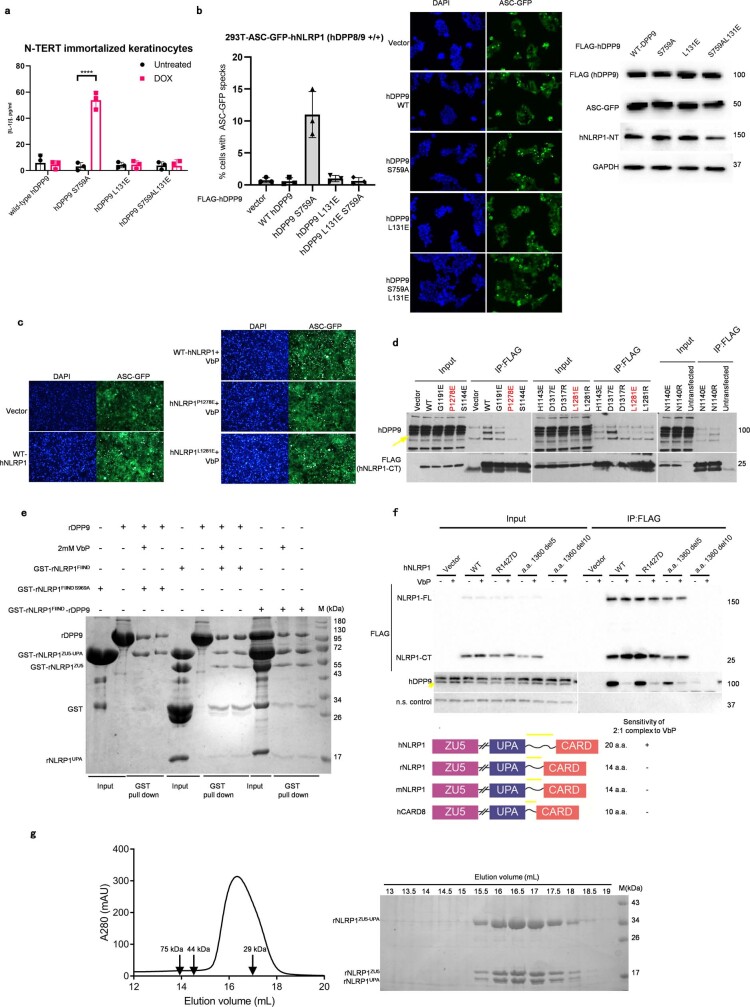
Additional evidence for the supression of NLRP1 by DPP9 binding and enzymatic activity. **a**, Immortalized keratinocytes stably transduced with Tet-ON-3×Flag hDPP9 lentiviruses were treated with or without doxycycline for 24 h. Conditioned media were subjected to anti-IL-1β ELISA. The *y* axis represents the concentrations of IL-1β in the medium. Bar graphs represent data from three inductions. Two-way ANOVA, ****P < 0.0001. **b**, Middle, representative images of 293T cells expressing ASC–GFP and hNLRP1 and transfected with wild-type hDPP9 or hDPP9 mutants. Left, the percentage of cells with ASC–GFP specks was quantified using more than 200 cells (left). Right, the expression of wild-type hDPP9 or hDPP9 mutants in 293T cells expressing ASC–GFP and NLRP1. The soluble lysate was blotted for hDPP9, hNLRP1-NT, ASC–GFP or GAPDH. Each experiment was repeated at least three times. **c**, Representative images of ASC–GFP expressing 293T cells transfected with the indicated hNLRP1 mutants. VbP (3 μM) was added 24 h post transfection. Cells were fixed 48 h post transfection and the nuclei were counterstained with Hoescht 33342. The percentage of cells with ASC–GFP specks was quantified using more than 200 cells. **d**, ASC–GFP 293T cells transfected with C-terminally Flag-tagged hNLRP1 constructs. Anti-Flag immunoprecipitation was performed on approximately 1 mg whole-cell lysate 48 h after transfection. **e**, GST-fused rNLRP1 FIIND and rDPP9 were individually purified from insect cells. Wild-type or mutant rNLRP1 FIIND proteins were used to pull down rDPP9 with GS4B resin in the presence or in the absence of VbP. **f**, Top, 293T cells were transfected with the indicated hNLRP1–Flag mutants. Anti-Flag immunoprecipitation was performed as in **d**. The hDPP9 band is indicated with the yellow arrow. The linker region between hNLRP1 UPA and CARD begins at amino acid 1360. Bottom, comparison of the linker regions of hNLRP1, rNLRP1, NLRP1B and hCARD8. The linker length and the sensitivity of the respective 2:1 complex to VbP are shown on the right. **g**, The gel filtration profile (left) and SDS–PAGE (right) of the rNLRP1 FIIND. See Supplementary Figs. 2 and 5 for gel raw data. Source data

rNLRP1-bound rDPP9 displayed protease activity, which was inhibited by VbP (Extended Data Fig. 7f). The hDPP9(S759A)–hNLRP1 complex is therefore biochemically similar to VbP-bound hDPP9–hNLRP1. Overexpression of hDPP9(S759A) was expected to outcompete endogenous hDPP9 for hNLRP1 binding to form the hDPP9(S759A)–hNLRP1 complex, which is defective in hNLRP1 inhibition. Consistent with our previous results^26^, VbP treatment substantially induced the secretion of mature IL-1β (molecular mass 17 kDa) and extensive death of human keratinocytes (Fig. 3e, f, Extended Data Fig. 8a), indicating that caspase-1 had been activated in these cells. Supporting our prediction, the doxycycline-induced expression of hDPP9(S759A)—but not of the hNLRP1-binding-deficient hDPP9(L131E) or hDPP9(S759A/L131E) mutants—in these cells caused IL-1β secretion and pyroptosis (Fig. 3e, f, Extended Data Fig. 8a). Similar observations were also made using 293T cells expressing ASC–GFP and NLRP1 (DPP8^+^DPP9^+^) (Extended Data Fig. 8b). These data further support the dual requirement of the catalytic function of hDPP9 and its direct binding to hNLRP1 in order to suppress the activation of hNLRP1 in human cells.

## Inhibition of UPA–CARD oligomerization by ZU5

**Fig. 4:**
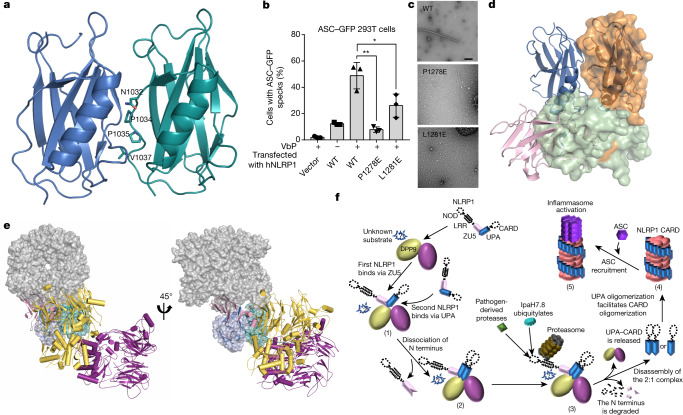
Mechanism of ZU5-mediated NLRP1 inhibition by DPP9. **a**, Cartoon representation of the UPA dimer in the 2:1 rNLRP1–rDPP9 complex. Key residues that mediate the formation of the dimeric UPA are shown in stick representation. **b**, The effect of the mutants hNLRP1(P1278E) and hNLRP1(L1281E) on the VbP-induced activation of hNLRP1 in 293T cells. Bar graphs represent data from three inductions. Student’s *t*-test, *P < 0.05, **P < 0.01. **c**, Negative staining electron microscopy analysis of human wild-type and mutant UPA–CARD fragments. Scale bar, 100 nm. **d**, Alignment of the UPA homodimer from the 2:1 rNLRP1–rDPP9 complex (in cartoon) with the crystal structure of rNLRP1 FIIND (shown as a transparent surface). **e**, Structural alignment of the oligomeric UPA–CARD fragment (shown in surface representation, PDB ID: 6K7V) with the rNLRP1–rDPP9 complex. The two UPA molecules (in blue and cyan) from the complex were used as the template for alignment. **f**, Working model of the DPP9-mediated inhibition of NLRP1 and the pathogen-induced activation of NLRP1. In resting cells, an autoinhibited rNLRP1 interacts with a dimeric rDPP9 via its autoinhibitory ZU5 domain (1). This interaction enables rDPP9 to recruit an autocleaved NLRP1, resulting in dissociation of the N-terminal segment from the C-terminal UPA–CARD fragment and formation of a 2:1 rNLRP1–rDPP9 complex (2). The two UPA–CARD fragments in the complex are sequestered from oligomerization by interaction with the active sites of rDPP9 and interaction with the DPP9-bound autoinhibited rNLRP1 via UPA–UPA dimerization. There may exist a DPP9 substrate that has a role in NLRP1 inhibition, because protease activity is important in order for rDPP9 to suppress NLRP1. Such a substrate could also have a role in NLRP1 activation in the absence of DPP9. Pathogen-induced proteasomal degradation would lead to the release of the active UPA–CARD fragments from the complex (3). The released UPA–CARD fragment then oligomerizes (4) to recruit ASC for the activation of downstream immune signalling (5). Source data

The residues N1032, P1034, P1035 and V1037, at the centre of the UPA–UPA surface (Fig. 4a), are conserved among NLRP1 proteins from different species and CARD8 (Extended Data Fig. 1d). Notably, hNLRP1-2, an hNLRP1 splice isoform that lacks this loop region, displayed impaired cell-killing activity when ectopically expressed in MCF7 cells^27^, supporting an essential role of this loop in hNLRP1-mediated cell death. Further supporting this conclusion, mutations of P1278 and L1281 of hNLRP1—from the corresponding UPA dimerization interface—substantially reduced VbP-induced ASC speck formation in ASC–GFP-expressing 293T cells (Fig. 4b, Extended Data Fig. 8c, d).

UPA forms a ring-like oligomer, which then brings the CARDs into close proximity for the efficient, filament-like polymerization of hNLRP1 UPA–CARD^28^. The UPA–UPA interaction surface observed in the 2:1 NLRP1–DPP9 complex might be similar to that in the UPA–CARD filament. Indeed, the wild-type UPA–CARD fragment of hNLRP1 has been found to form filamentous structures^28,29^, but the UPA–UPA interface mutants—UPA–CARD(P1278E) and UPA–CARD(L1281E)—did not (Fig. 4c), which indicates that the UPA–UPA dimer interface is required for higher-order UPA oligomerization and activation of the hNLRP1 inflammasome.

VbP had little effect on the interaction of the rNLRP1 FIIND with rDPP9 (Extended Data Fig. 8e), which is consistent with data from NLRP1B and CARD8 but contrasts with those from hNLRP1 (refs. ^8,9^). It is of interest to note that the linker region between UPA and CARD in hNLRP1 is longer than that in NLRP1B and CARD8 (Extended Data Figs. 1d, 8f). Deletion analysis suggested a role of the linker region in the sensitivity of rDPP9-mediated hNLRP1 inhibition to VbP (Extended Data Fig. 8f). The mutation hNLRP1(P1214R), which is associated with autoinflammatory diseases, could perturb the interaction of hNLRP1 with hDPP9 at the UPA-binding site^9^.

In contrast to free UPA^28,29^, the FIIND was monomeric in solution (Extended Data Fig. 8g), which suggests that the ZU5 domain inhibits UPA dimerization or oligomerization. Supporting this notion, structural comparison revealed that ZU5 in the monomeric FIIND sterically hinders UPA dimerization in the 2:1 complex (Fig. 4d). In its activated state, the UPA–CARD fragment forms helical filaments in which dimeric UPA spirally wraps around the inner CARDs^28^. Alignment of the dimeric UPA from the 2:1 rNLRP1–rDPP9 complex with that from the UPA–CARD filament^28^ showed that ZU5 is positioned to block the spiral growth of the dimeric UPA (Fig. 4e). Collectively, these results show that ZU5 negatively regulates NLRP1 activation by directly or indirectly inhibiting the formation of UPA–CARD filaments.

## Discussion

Sequestration of the potent UPA–CARD fragment in the 2:1 rNLRP1–rDPP9 complex can block the UPA-mediated formation of functional UPA–CARD filaments (Fig. 4f). This is consistent with the idea that sequestration of active domains of NLRs is a general strategy in the regulation of inflammasomes^30^. Our data suggested that the ZU5 domain is also important for the inhibition of DPP9-independent UPA–CARD activation (Fig. 4d, Extended Data Fig. 8g). The ZU5 domain therefore seems to be critical for the negative regulation of both DPP9-independent and DPP9-dependent NLRP1 activation. The degradation of N-terminal fragments of NLRP1—induced by either lethal toxin or 3C proteases or by chemical inhibition of DPP9—can similarly disrupt ZU5-mediated interactions and consequently release the autoinhibited and the sequestered UPA–CARD fragments in the 2:1 NLRP1–DPP9 complex (Fig. 4f). Thus, through disruption of the ZU5-dependent interaction between NLRP1 and DPP9, N-terminal degradation of NLRP1 is the unifying mechanism of NLRP1 activation^1–3,14^.

It remains unknown why the protease activity of DPP9 is important for NLRP1 inhibition. A plausible explanation is the existence of a substrate(s) that is required for inhibition, but how the substrate is involved is unclear. The requirement for protease activity raises the possibility that perturbations of this activity by pathogen-derived or host-derived molecules can trigger NLRP1 activation. If this is the case, the 2:1 NLRP1–DPP9 complex could represent a bona fide receptor that senses diverse signals that induce the N-terminal degradation of NLRP1 or perturb the protease activity of DPP9.

## Methods

### Data reporting

No statistical methods were used to predetermine sample size. The experiments were not randomized and the investigators were not blinded to allocation during experiments and outcome assessment.

### Protein expression and purification

The genes encoding full-length rNLRP1(GenBank ID: HM060632.1) and full-length rDPP9 (NCBI Reference Sequence: NM_001305241.1) were synthesized by Genewiz. The constructs of rNLRP1 (residues 1–1218), rDPP9 (residues 1–862, wild-type and all mutants), rNLRP1 FIIND (residues 822–1122, wild-type and all mutants) and rNLRP1 FIIND–CARD (residues 822–1218) were generated by a standard PCR-based cloning strategy and cloned into pFastBac-1 vector with an N-terminal GST tag or with no tag, and their identities were confirmed by sequencing. All the proteins were expressed using the Bac-to-Bac baculovirus expression system (Invitrogen) in sf21 cells at 28 °C. One litre of cells (2.5 × 10^6^ cells per ml, medium from Expression Systems) was infected with 20 ml baculovirus at 28 °C. After growth at 28 °C for 48 h, the cells were collected, resuspended in the buffer containing 25 mM Tris-HCl pH 8.0 and 150 mM NaCl, and lysed by sonication. The soluble fraction was purified from the cell lysate using Glutathione Sepharose 4B beads (GS4B, GE Healthcare). The proteins were then digested with PreScission protease (GE Healthcare) to remove the GST tag and further purified by gel filtration (SuperoseTM 6 prep grade XK 16/70; GE Healthcare). To prepare the rNLRP1 FIIND for crystallization trials, the purified rNLRP1 FIIND (residues 822–1122) was concentrated to about 8.0 mg ml^−1^ in buffer containing 100 mM NaCl, 10 mM Tris-HCl pH 8.0. For co-expression of rNLRP1 and rDPP9, one litre of sf21 cells were co-infected with 10 ml recombinant baculovirus of rNLRP1 and rDPP9, and then the rNLRP1–rDPP9 complex was purified using GS4B beads. Similar protocols were used to purify the complex containing full-length GST–CARD8 and hDPP9. For cryo-EM investigation, the purified rNLRP1–rDPP9 complex was concentrated to about 0.3 mg ml^−1^ in buffer containing 25 mM Tris-HCl pH 8.0, 150 mM NaCl and 3 mM DTT.

Recombinant hNLRP1 UPA–CARD tagged with a removable Snap domain was expressed using bacterial vectors as the form of inclusion bodies. After cellular lysis, the cellular pellet was collected after centrifugation at 30,000*g* for 30 min at 4 °C. Several additional washes using wash buffer (20 mM Tris-HCl, pH 8.0, 150 mM NaCl, 1% Triton-X and 1 mM DTT) were performed until a pure white pellet was obtained. The pellet was dissolved in 6 M guanidinium, and centrifuged at 30,000*g* for a second time for 30 min at room temperature to remove contaminants. The denatured soluble proteins were then gradually dialysed against 3, 2, 1.5, 1, 0.8 and 0.6 M guanidinium in dialysis buffer (20 mM Tris-HCl, pH 8.0, 150 mM NaCl, 5 mM β-mercaptoethanol) in the cold room, and eventually in fresh dialysis buffer without guanidinium. The refolded proteins were centrifuged for a third time at 10,000*g* for 10 min to remove misfolded aggregates. The soluble refolded fractions were then subjected to biochemical analysis and negative-stain electron microscopy experiments. The Snap tag was removed by 3C proteases, and the product was further purified by reverse Ni-NTA purification.

### Gel-filtration assay

The GST–rNLRP1 FIIND(S969A)–rDPP9 complex and rNLRP1 FIIND proteins purified as described in the previous section were subjected to gel filtration (Superose 6, 10/30; GE Healthcare) in buffer containing 10 mM Tris pH 8.0 and 100 mM NaCl. The purified rNLRP1 FIIND was left at 18 °C for two weeks to obtain its fully autoprocessed form. The fully processed rNLRP1 FIIND was then incubated with the purified GST–rNLRP1 FIIND(S969A)–rDPP9 complex at a molar ratio of about 1:1 in 4 °C for 150 min before gel-filtration analysis. Samples from relevant fractions were applied to an SDS–PAGE gel and visualized by Coomassie blue staining. A similar procedure was used to assay the interaction of rDPP9 with other rNLRP1 mutant proteins.

### Pull-down assay

Sf21 cells (50 ml; 2.5 × 10^6^ cells per ml, medium from Expression Systems) were infected with 1 ml baculovirus of GST–rNLRP1 FIIND (wild-type or mutants), and the proteins were expressed and purified as described in the section ‘Protein expression and purification’. In brief, the proteins were purified from the cell lysate using 300 μl GS4B resin (GS4B, GE Healthcare), and incubated with an excess of purified wild-type or mutant rDPP9 proteins on ice for 60 min. The resin was washed with 1 ml buffer containing 10 mM Tris pH 8.0, 100 mM NaCl five times, and eluted with 300 μl buffer containing 25 mM Tris pH 8.0, 150 mM NaCl, 15 mM GSH. The eluted samples were analysed by SDS–PAGE and visualized by Coomassie blue staining. A similar procedure was used to assay the interaction between full-length CARD8 and hDPP9.

To test the effect of VbP on the rNLRP1–rDPP9 interaction, 2 mM VbP was added to the purified rDPP9, GST–rNLRP1(S969A)–rDPP9 complex or GST–rNLRP1 FIIND–rDPP9 complex. After 60-min incubation, the samples were individually incubated with wild-type or mutant rNLRP1 FIIND and 100 μl GS4B resin on ice for 60 min. After extensive washing, the proteins bound in the resin were eluted and analysed by SDS–PAGE and visualized by Coomassie blue staining.

### Enzymatic activity assay

To measure rDPP9 protease activity, a stock solution of substrate (10 mM Gly-Pro-AMC) was prepared in DMSO. Purified wild-type or mutant rDPP9 was diluted to 1 μM to a final volume of 100 μl in buffer containing 10 mM Tris pH 8.0 and 100 mM NaCl. The substrate Gly-Pro-AMC (10 μl of 10 μM solution, dissolved in DMSO) was added to the mixture. Substrate cleavage was measured by the liberated AMC fluorescence signal recorded at room temperature in a luminescence spectrometer at excitation and emission wavelengths of 380 nm and 500 nm, respectively, over a period of 30 min.

To measure the protease activity of hDPP9, 293T cells were transfected with hDPP9 and lysed 48 h post transfection in 1× Tris-buffered saline (TBS) with 0.25% NP40. Lysate (0.3 μg) was mixed with 0.1 μl 100 mM Gly-Pro-AMC in 50 μl lysis buffer. AMC fluorescence (380 nm excitation; 500 nm emission) was monitored at room temperature for 30 min at 1-min intervals.

### Edman degradation by the PPSQ-33A system

The phenylthiohydantoin amino acid was separated in the reversed-phase mode of high-performance liquid chromatography using the differences between the retention times of different amino acids, and the amount of UV absorbance at specific wavelengths was detected. The samples were transferred to the PVDF membrane and five cycles were set. The amino acid sequences of each sample were determined from the chromatograms obtained in each cycle evaluation performed by comparing chromatograms with those in the previous and subsequent cycles and identifying the phenylthiohydantoin amino acids that had the greatest increase in abundance.

### Cryo-EM sample preparation and data collection

An aliquot of 3 μl of purified rNLRP1–rDPP9 or rNLRP1 FIIND–CARD(S969A)–rDPP9 complex was applied to holey carbon grids (Quantifoil Au 1.2/1.3, 300 mesh), which were glow-discharged for 30 s at middle level in Harrick Plasma after 2 min evacuation. The grids were then blotted by filter papers (Ted Pella) for 2.5 s at 8 °C and 100% humidity, then flash-frozen in liquid ethane using FEI Vitrobot Marke IV.

Cryo-EM data for rNLRP1–rDPP9 and rNLRP1 FIIND–CARD(S969A)–rDPP9 were collected on a Titan Krios electron microscope operated at 300 kV, equipped with a Gatan K2 Summit direct electron detector and a Gatan Quantum energy filter (an additional Cs-corrector that was used for rNLRP1 FIIND–CARD(S969A)–rDPP9 data collection). A total of 7,157 and 4,971 micrograph stacks were automatically recorded using AutoEMation in super-resolution mode for rNLRP1–rDPP9 and rNLRP1 FIIND–CARD(S969A)–rDPP9, at a nominal magnification of 130,000× and 105,000×, respectively. Defocus values varied from −1.0 μm to −2.0 μm for both datasets^31^. Dose rates during the collection of data for rNLRP1–rDPP9 and rNLRP1 FIIND–CARD(S969A)–rDPP9 were 10 and 11 electrons per pixel per second, respectively. For both datasets, the exposure time of 5.6 s was dose-fractionated into 32 sub-frames, leading to a total accumulated dose of approximate 50 electrons per Å^2^ for each stack.

### Image processing and 3D reconstruction

The stacks of rNLRP1–rDPP9 and rNLRP1 FIIND–CARD(S969A)–rDPP9 recorded in super-resolution mode were motion-corrected using MotionCor2 and binned twofold, resulting in a physical pixel size of 1.061 Å per pixel and 1.091 Å per pixel, respectively^32^. Meanwhile, dose weighting for the summed micrographs was performed^33^. CTFFIND4 was then used to estimate the contrast transfer function (CTF) parameters^34^. On the basis of the CTF estimation, 7,033 and 4,667 micrographs were manually selected for rNLRP1–rDPP9 and rNLRP1 FIIND–CARD(S969A)–rDPP9, respectively, and were further processed in Relion 3.1. Approximately 2,000 particles were manually picked and 2D-classified to generate initial templates for autopicking. In the end, 2,700,586 and 1,725,380 particles were automatically picked for rNLRP1–rDPP9 and rNLRP1 FIIND–CARD(S969A)–rDPP9, respectively, using Relion 3.1. After several rounds of reference-free 2D classification, 1,430,734 particles for rNLRP1–rDPP9 and 1,117,656 particles for rNLRP1 FIIND–CARD(S969A)–rDPP9 were subjected to 3D classification, using the initial 3D reference models obtained by ab initio calculation from Relion 3.1. Particles from good 3D classes, with better overall structure features, were selected for 3D refinement. After global 3D refinement and post-processing, the resolution was 3.07 Å with a particle number of 343,648 for rNLRP1–rDPP9, and 3.69 Å with a particle number of 252,425 for rNLRP1 FIIND–CARD(S969A)–rDPP9.

To improve the quality of the density of the NLRP1 section in the rNLRP1–rDPP9 map, the rNLRP1–rDPP9 particles after 3D refinement were then subjected to a further round of focused 3D classification with a local mask generated using Chimera. A previously reported focused 3D classification procedure was adopted to select the 3D class with good density^35^. Ultimately, a subset of 182,116 particles after focused 3D classification were subjected to a final 3D refinement and yielded a global reconstruction at 3.18 Å after postprocess.

2D classification, 3D classification and 3D autorefinement were all performed using Relion 3.1 (refs. ^36–38^). The resolutions were determined by gold-standard Fourier shell correlation^39^. Local resolution distribution was evaluated^40^ using Relion 3.1.

### Crystallization, data collection and structure determination

Crystallization of rNLRP1 was performed by hanging-drop vapour-diffusion methods, mixing 1 μl of 8 mg ml^−1^ protein with 1 μl of reservoir solution at 18 °C. Good-quality crystals of rNLRP1 FIIND were obtained in buffer containing 1.0 M ammonium sulfate, 0.1 M Bis-Tris pH 5.5, 1% w/v polyethylene glycol 3,350. All the crystals were flash-frozen in reservoir buffer to which glycerol (15%) was added as the cryo-protectant to prevent radiation damage. The diffraction dataset was collected at the Shanghai Synchrotron Radiation Facility (SSRF) on the beamline BL19U1 using a CCD detector and was processed using HKL2000 software package. The crystal structure of rNLRP1 FIIND was determined by PHASER_MR with the structure of NUC5b as the search model. The model from the molecular replacement was manually rebuilt to the sequence of rNLRP1 FIIND in the program Coot^41^ and subsequently subjected to refinement by the program Refine_Phenix^42^. Data collection, processing, and refinement statistics are summarized in Extended Data Table 1.

### Model building and refinement

The EM density map of rNLRP1–rDPP9 was used for model building, as the quality of density for rNLRP1 was sufficient for sequence assignment. The model of hDPP9 (PDB ID: 6EOQ)^23^, along with two copies of the rNLRP1 FIIND crystal structure that we determined as described in the previous section, were docked into the EM density map of rNLRP1–rDPP9 in Chimera^43^. The sequence of hDPP9 was changed to that of rDPP9, the whole model containing two rNLRP1 FIIND molecules and a rDPP9 dimer was then adjusted manually in the program Coot^41^, and refined against the EM map by Phenix in real space with secondary structure and geometry restraints^42^. The final model of the rNLRP1–rDPP9 complex was validated using MolProbity and EMRinger in the Phenix package^42^. The model statistics are summarized in Extended Data Table 2.

### ASC–GFP transfection in 293T cells and ASC-GFP speck formation assay

293T ASC–GFP and 293T ASC–GFP DPP8/DPP9 double-knockout cells have been previously described^9^. All transfections were carried out using Lipofectamine 2000 (Thermo Fisher). For immunoprecipitation, cells were collected 48 h post transfection. For the ASC–GFP speck assay, cells were fixed 24 h post transfection and counterstained with DAPI or Hoescht before wide-field fluorescence imaging. The number of nuclei per field of view was counted in ImageJ using the following image processing steps: ‘Threshold’ (20–30 to 255); ‘Watershed’; and ‘Analyze Particles’ (200–infinity). ASC specks were counted in ImageJ in the GFP channel using ‘Find Maxima’ (prominence = 20).

### Inflammasome activation assays in immortalized keratinocytes

Immortalized human keratinocytes (N/TERT-1) were a gift from H. Reinwald (Harvard University) (Material Transfer Agreement to Skin Research Institute of Singapore). Stably transduced N/TERT-1 cells were induced with doxycycline (1 μg ml^−1^) for 24 h. Immunoblotting antibodies used were as follows: anti-IL-1β p17 specific (CST, 83186S); GAPDH (Santa Cruz Biotechnology, sc-47724); IL-1β (R&D systems, AF-201); anti-Flag tag (Sigma Aldrich, F3165). All horseradish peroxidase (HRP)-conjugated secondary antibodies were purchased from Jackson Immunoresearch (goat anti-mouse IgG, 115-035-166; goat anti-rabbit IgG, 111-035-144; and donkey anti-goat IgG, 705-005-147).

### Reporting summary

Further information on research design is available in the Nature Research Reporting Summary linked to this paper.

## Online content

Any methods, additional references, Nature Research reporting summaries, source data, extended data, supplementary information, acknowledgements, peer review information; details of author contributions and competing interests; and statements of data and code availability are available at 10.1038/s41586-021-03320-w.

### Supplementary information

Supplementary Figures This file contains Supplementary Figures 1-5 displaying the uncropped scans.

Reporting Summary

### Source data

Source Data Fig. 3

Source Data Fig. 4

Source Data Extended Data Fig. 7

Source Data Extended Data Fig. 8

## Figures and Tables

**Extended Data Table 1:**
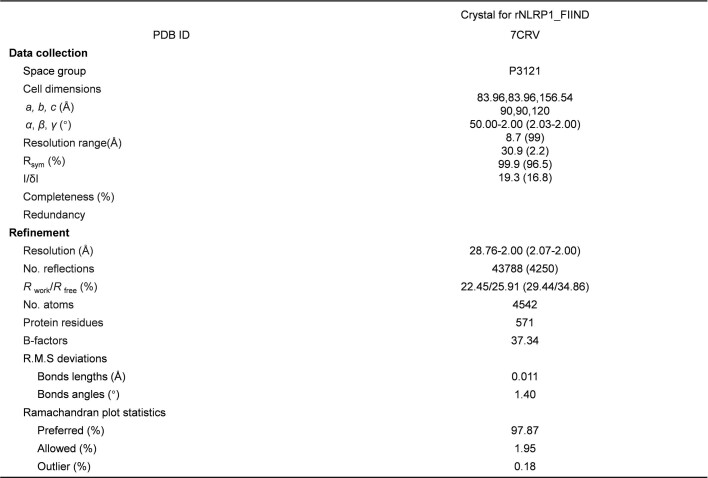
Crystallography data collection and refinement statistics

**Extended Data Table 2:**
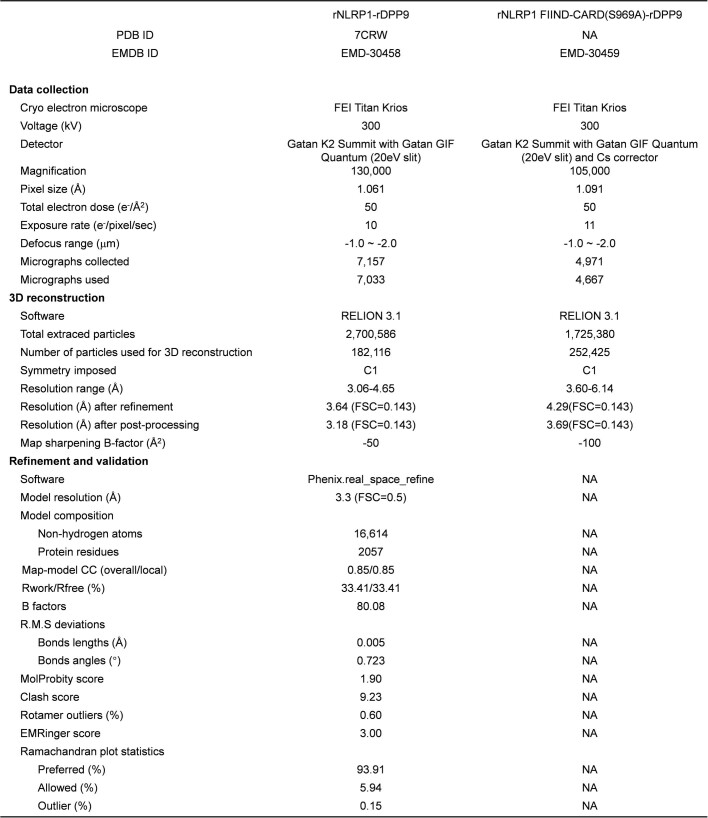
Cryo-EM data collection, 3D reconstruction and model statistics

## Data Availability

The atomic coordinates and structure factors have been deposited in the RCSB Protein Data Bank (PDB) and Electron Microscopy Data Bank (EMDB). The PDB codes of the rNLRP1 FIIND and rNLRP1–rDPP9 structures are 7CRV and 7CRW, respectively. The EMDB codes of the rNLRP1–rDPP9 and rNLRP1 FIIND–CARD(S969A)–rDPP9 structures are EMD-30458 and EMD-30459, respectively. For gel source images, see Supplementary Information. All other data or materials can be obtained from the corresponding author upon request. Source data are provided with this paper.
