# Supplementary material for: Structural and biochemical mechanisms of NLRP1 inhibition by DPP9
Source: Nature. 2021 Mar 17;592(7856):773–7. doi: 10.1038/s41586-021-03320-w (PMC8081665; doi:10.1038/s41586-021-03320-w)
Supplement: Supplementary file 1 — This file contains Supplementary Figures 1-5 displaying the uncropped scans. [file 41586_2021_3320_MOESM1_ESM.pdf]

nature portfolio

https://doi.org/10.1038/s41586-021-03320-w

Supplementary information

Structural and biochemical mechanisms of NLRP1 inhibition by DPP9

In the format provided by the authors and unedited

Structural and biochemical mechanisms of NLRP1 inhibition by DPP9

Menghang Huang[1, #], Xiaoxiao Zhang[1, #], Toh Gee Ann[2], Qin Gong[4], Jia Wang[1], Zhifu Han[1], Bin Wu[4,5], Franklin Zhong[2,3,*] and Jijie Chai[1,6,7,*]

[1]Beijing Advanced Innovation Center for Structural Biology, Tsinghua- Peking Joint Center for Life Sciences, Center for Plant Biology, School of Life Sciences, Tsinghua University, 100084 Beijing, China

[2]Lee Kong Chian School of Medicine, Nanyang Technological University, 11 Mandalay Road, 308232, Singapore

[3]Skin Research Institute of Singapore (SRIS), 8A Biomedical Grove, #06-06 Immunos, 138648, Singapore

[4]School of Biological Sciences, Nanyang Technological University

[5]Institute of Structural Biology, Nanyang Technological University

[6]Max Planck Institute for Plant Breeding Research, 50829 Cologne, Germany

[7]Institute of Biochemistry, University of Cologne, Zuelpicher Strasse 47, 50674 Cologne, Germany

[#]Equal contributions

*To whom correspondence should be addressed: chaijj@mail.tsinghua.edu.cn or Franklin.zhong@ntu.edu.sg

Figure 1d

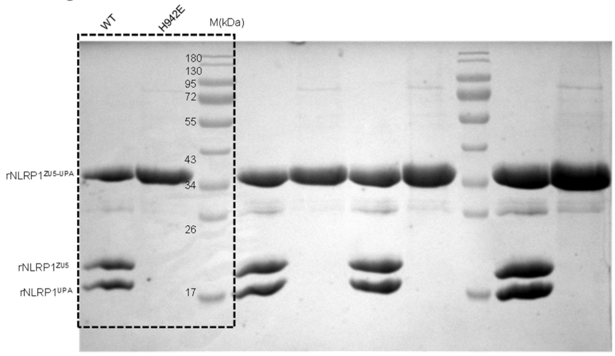

Figure 2e

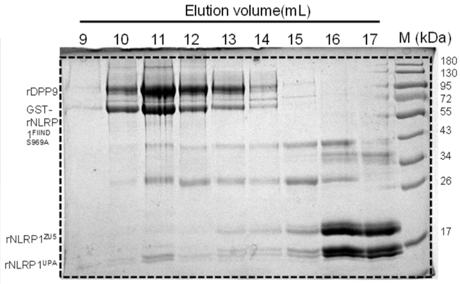

Figure 3a

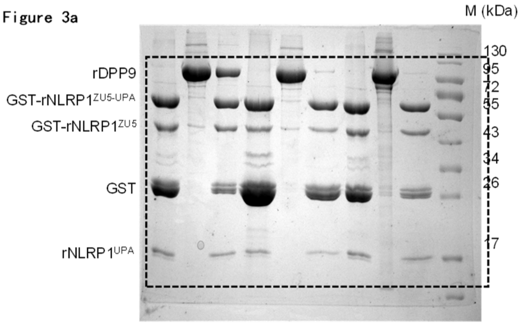

Extended Data Figure 1b

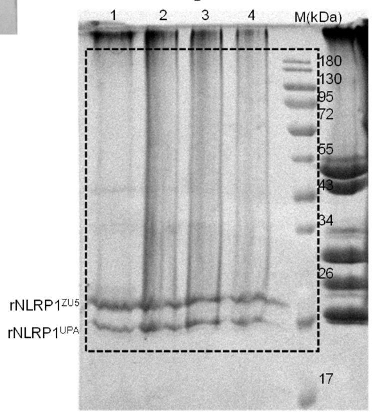

Extended Data Figure 2a

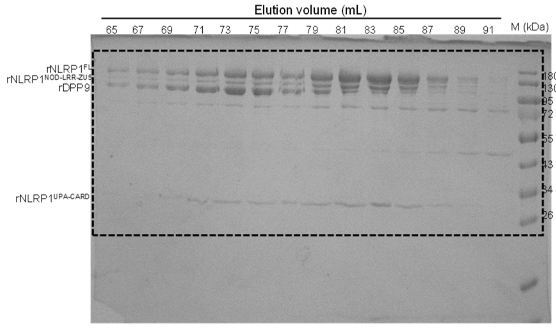

Extended Data Figure 2b

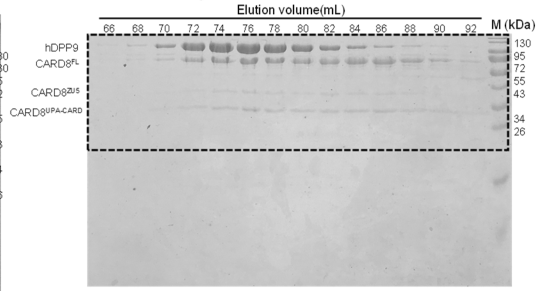

Extended Data Figure 5b

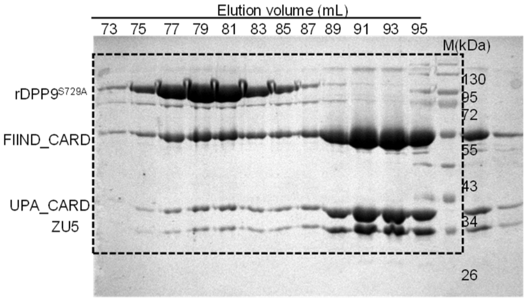

Extended Data Figure 5c

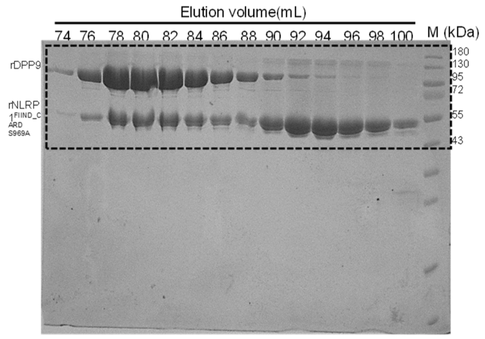

Supplementary Figure 1 | Full scans of commassie stained gels presented in Figure. 1d, 2e, 3a and Extended Data Figure 1b, 2a, 2b, 5b, 5c.

The frames surround areas that were cropped to assemble the main figures**.** The molecular weight of the ladder of each gel was marked.

Extended Data Figure 7a

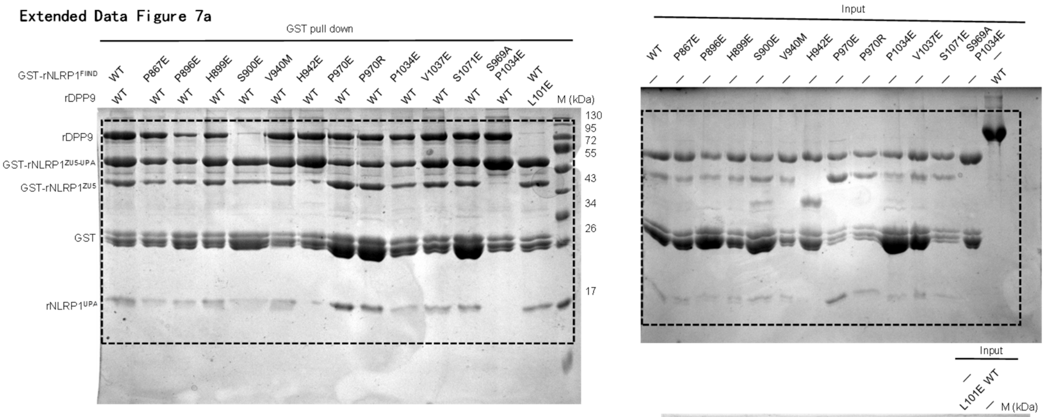

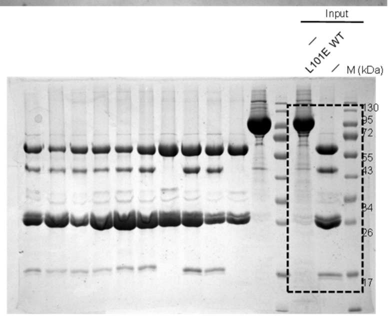

Extended Data Figure 7b

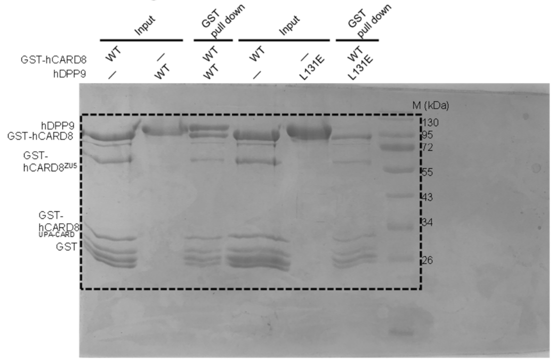

Extended Data Figure 7f

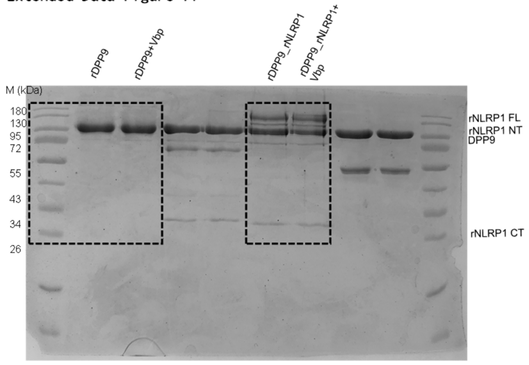

Extended Data Figure 8e

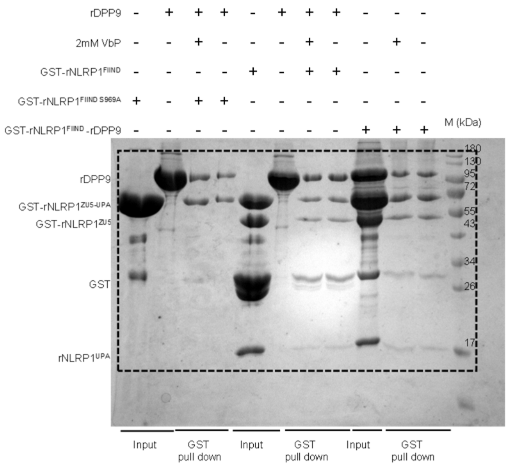

Extended Data Figure 8g

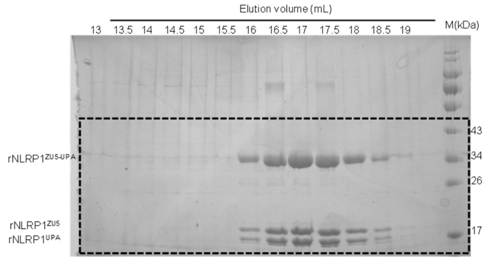

Supplementary Figure 2 | Full scans of commassie stained gels presented in Extended Data Fig. 7a, 7b, 7f, 8e and 8g.

The frames surround areas that were cropped to assemble the main figures**.** The molecular weight of the ladder of each gel was marked.

Figure 3b

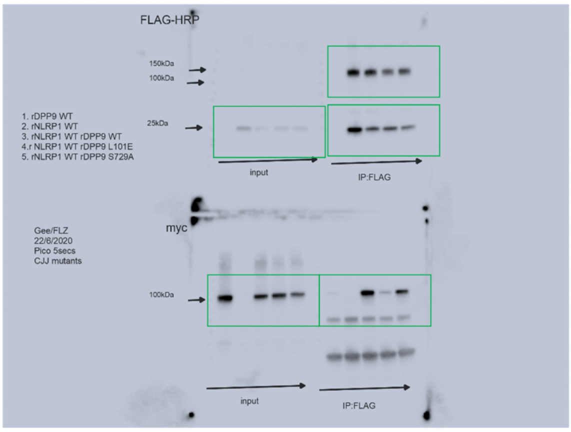

Figure 3c

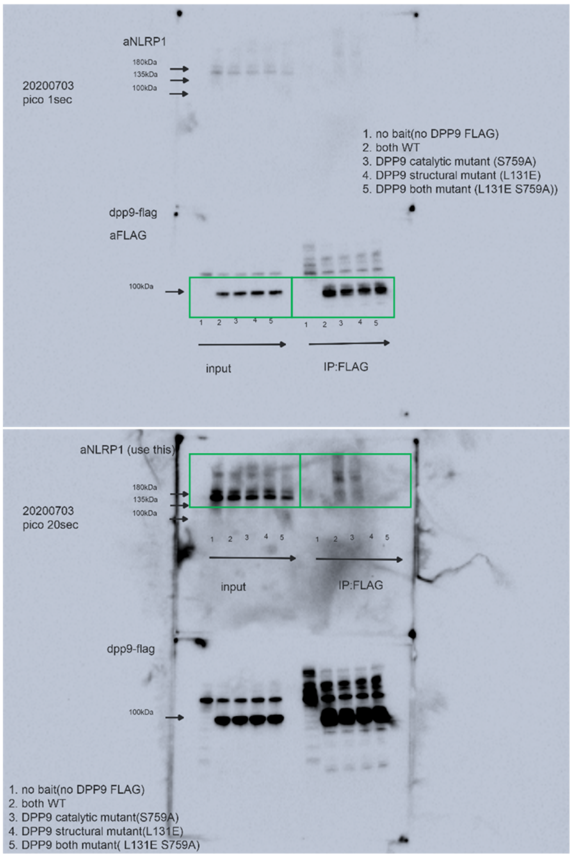

Figure 3e

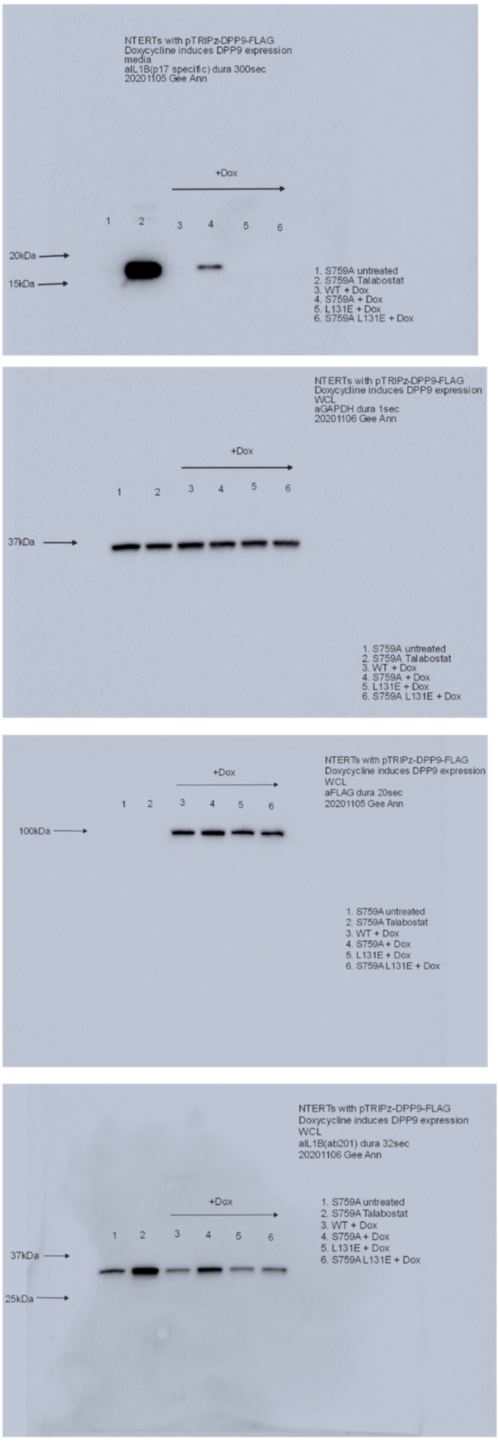

Supplementary Figure 3 | Full scans of western blots presented in Fig. 3b, 3c and 3e.

The frames surround areas that were cropped to assemble the main figures**.** The molecular weight of the ladder of each gel was marked.

Extended Data Figure 7c

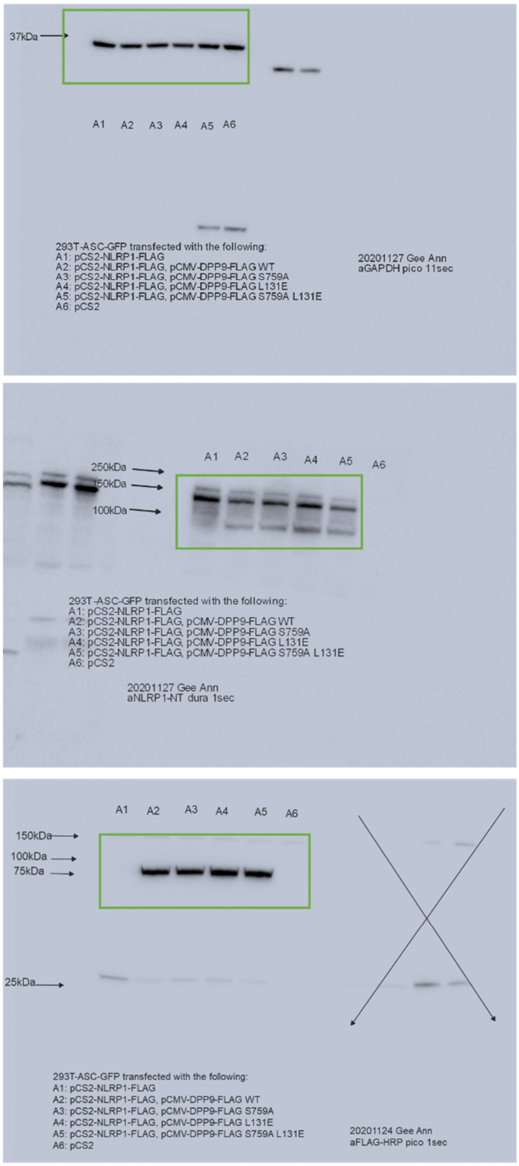

Extended Data Figure 7e

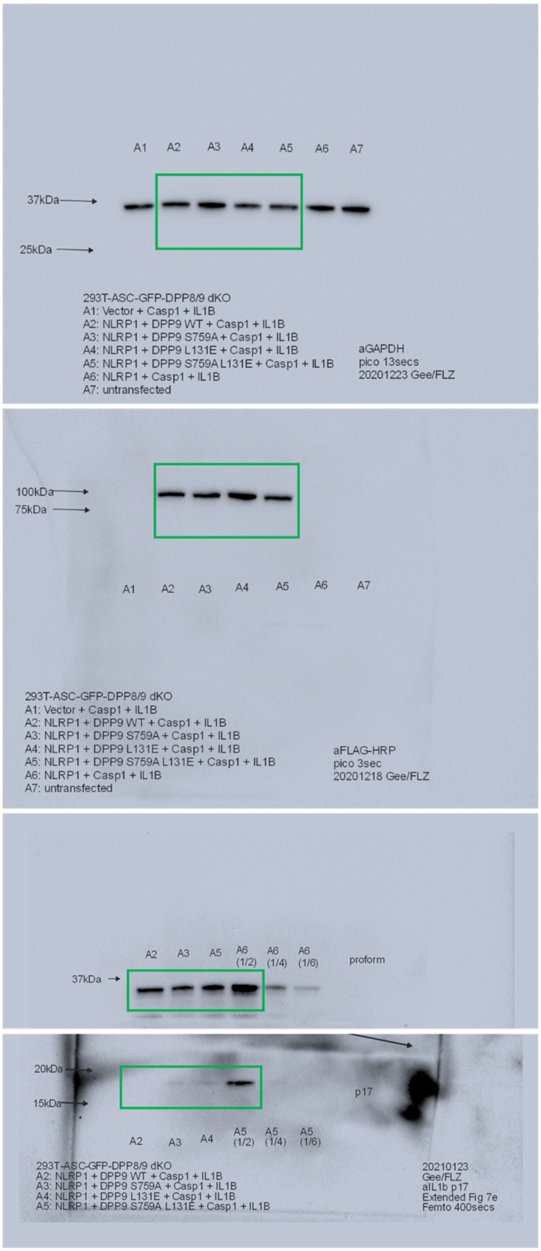

Supplementary Figure 4 | Full scans of western blots presented in Extended Data Fig. 7c and 7e.

The frames surround areas that were cropped to assemble the main figures**.** The molecular weight of the ladder of each gel was marked.

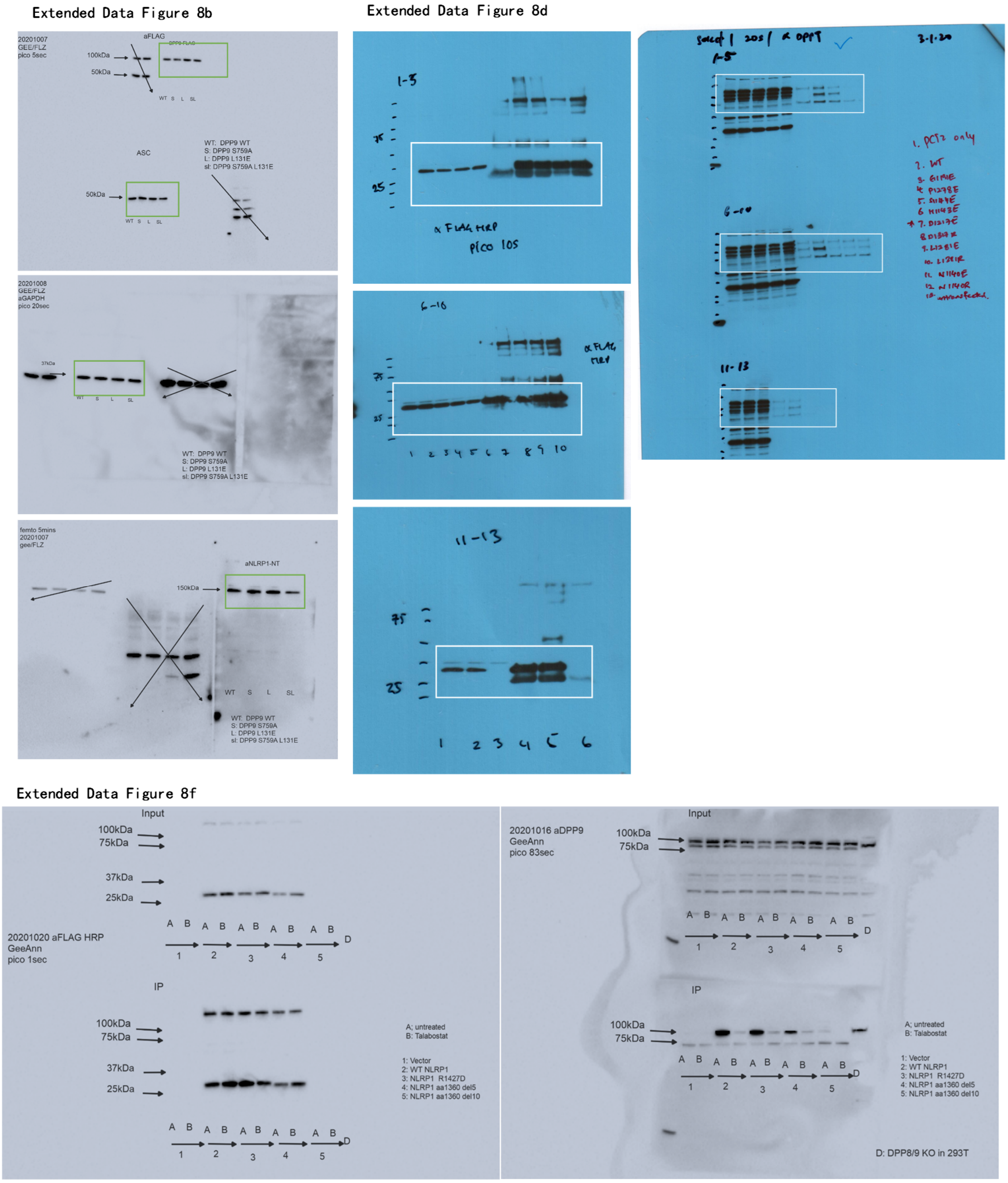

Supplementary Figure 5 | Full scans of western blots presented in Extended Data Fig. 8b, 8d and 8f.

The frames surround areas that were cropped to assemble the main figures**.** The molecular weight of the ladder of each gel was marked.
