# Supplementary material for: Structural and biochemical mechanisms of NLRP1 inhibition by DPP9
Source: Nature. 2021 Mar 17;592(7856):773–7. doi: 10.1038/s41586-021-03320-w (PMC8081665; doi:10.1038/s41586-021-03320-w)
Supplement: Supplementary file 2 — Reporting Summary [file 41586_2021_3320_MOESM2_ESM.pdf]

nature research

Corresponding author(s): Jijie Chai, Franklin Zhong

Last updated by author(s): Jan 25, 2021

Reporting Summary

Nature Research wishes to improve the reproducibility of the work that we publish. This form provides structure for consistency and transparency in reporting. For further information on Nature Research policies, see our Editorial Policies and the Editorial Policy Checklist.

Statistics

For all statistical analyses, confirm that the following items are present in the figure legend, table legend, main text, or Methods section.

n/a	Confirmed	
☐	☒	The exact sample size (n) for each experimental group/condition, given as a discrete number and unit of measurement
☐	☒	A statement on whether measurements were taken from distinct samples or whether the same sample was measured repeatedly
☐	☒	The statistical test(s) used AND whether they are one- or two-sided<br>*Only common tests should be described solely by name; describe more complex techniques in the Methods section.*
☒	☐	A description of all covariates tested
☐	☒	A description of any assumptions or corrections, such as tests of normality and adjustment for multiple comparisons
☐	☒	A full description of the statistical parameters including central tendency (e.g. means) or other basic estimates (e.g. regression coefficient) AND variation (e.g. standard deviation) or associated estimates of uncertainty (e.g. confidence intervals)
☐	☒	For null hypothesis testing, the test statistic (e.g. F, t, r) with confidence intervals, effect sizes, degrees of freedom and P value noted<br>*Give P values as exact values whenever suitable.*
☒	☐	For Bayesian analysis, information on the choice of priors and Markov chain Monte Carlo settings
☒	☐	For hierarchical and complex designs, identification of the appropriate level for tests and full reporting of outcomes
☒	☐	Estimates of effect sizes (e.g. Cohen's d, Pearson's r), indicating how they were calculated

Our web collection on statistics for biologists contains articles on many of the points above.

Software and code

Policy information about availability of computer code

Data collection	Blu-Ice BL19U1, AutoEmation.
Data analysis	RELION 3.1, MotionCor2 1.0.5, CTFFIND 4.1.12, CCP4i 7.1, Phenix 1.18, Chimera 1.14, Coot 0.8.9, Pymol 4.6.0, HKL2000 v718, Image J1.8.0

For manuscripts utilizing custom algorithms or software that are central to the research but not yet described in published literature, software must be made available to editors and reviewers. We strongly encourage code deposition in a community repository (e.g. GitHub). See the Nature Research guidelines for submitting code & software for further information.

Data

Policy information about availability of data

All manuscripts must include a data availability statement. This statement should provide the following information, where applicable:

- Accession codes, unique identifiers, or web links for publicly available datasets
- A list of figures that have associated raw data
- A description of any restrictions on data availability

All relevant data are available from the authors and/pr included in the manuscript of Supplementary Information. Atomic coordinates and the EM map have been deposited in the Protein Data Bank (PDB) and Electron Microscopy Data Bank (EMDB) under accession number 7CRW, 7CRV, EMD-30458 and EMD-30459.

Field-specific reporting

Please select the one below that is the best fit for your research. If you are not sure, read the appropriate sections before making your selection.

☒ Life sciences ☐ Behavioural & social sciences ☐ Ecological, evolutionary & environmental sciences

For a reference copy of the document with all sections, see nature.com/documents/nr-reporting-summary-flat.pdf

Life sciences study design

All studies must disclose on these points even when the disclosure is negative.

Sample size	For ASC speck assays, at least three transfections were carried out. A minimum of 100 cells were scored for the presence of ASC-GFP specks.
Data exclusions	Data exclusion for particles in bad 2D or 3D classes with no defined features was a standard processing for EM data.
Replication	To ensure reproducibility of experimental findings, each assay was performed at least three times to confirm the results.
Randomization	Animals or human research participants were not involved in this study and, as such, samples were not randomized for the experiments.
Blinding	Animals or human research participants were not involved in this study and, as such, samples were not blinded for the experiments.

Reporting for specific materials, systems and methods

We require information from authors about some types of materials, experimental systems and methods used in many studies. Here, indicate whether each material, system or method listed is relevant to your study. If you are not sure if a list item applies to your research, read the appropriate section before selecting a response.

Materials & experimental systems

n/a	Involved in the study	
☐	☒	Antibodies
☐	☒	Eukaryotic cell lines
☒	☐	Palaeontology and archaeology
☒	☐	Animals and other organisms
☒	☐	Human research participants
☒	☐	Clinical data
☒	☐	Dual use research of concern

Methods

n/a	Involved in the study	
☒	☐	ChIP-seq
☒	☐	Flow cytometry
☒	☐	MRI-based neuroimaging

Antibodies

Antibodies used	c-Myc Antibody (9E10): sc-40, Santa Cruz Biotechnology; FLAG antibody (M2), F3165, Sigma-Aldrich; Human NLRP1 antibody (AF6788), R&D systems;anti-IL-1β p17 specific (CST, #83186S);GAPDH (Santa Cruz Biotechnology, #sc-47724);IL-1β (R&D systems, #AF-201); anti-FLAG tag (SigmaAldrich, F3165).DPP9 antibody - Catalytic domain (Abcam, ab42080)<br>ASC antibody (Adipogen, AL177)<br>AffiniPure Goat Anti-Mouse IgG (H+L) (Code: 115-035-166,Jackson Immunoresearch)<br>Peroxidase AffiniPure Goat Anti-Rabbit IgG (H+L) (Code: 111-035-144,Jackson Immunoresearch)<br>All primary antibodies were used at 300ng/mL. All secondary antibodies were used at 1/7,500 (v/v).
Validation	All epitope tag and ASC antibodies had been validated by the manufacturers. NLRP1-NT antibody was validated using NLRP1-overexpressing 293T cells. DPP9 antibody had been previously validated using DPP8/9 knockout cells in Zhong et al, JBC, 2018.

Eukaryotic cell lines

Policy information about cell lines

Cell line source(s)	Sf21 cells lines from Thermo Fischer Scientific. https://www.thermofisher.com/order/catalog/product/12682019?SID=srch-srp-12682019#/12682019?SID=srch-srp-12682019.<br>293T (ATCC CRL-3216).<br>Immortalised human keratinocytes (N/TERT-1 or N-TERT herein) were provided by H. Rheinwald (MTA to Skin Research Institute of Singapore).
Authentication	Sf21 cell lines were verified by manufacturer's website and Identity of these cell lines were frequently checked by their morphological features. https://www.thermofisher.com/order/catalog/product/12682019?SID=srch-

	srp-12682019#/12682019?SID=srch-srp-12682019.<br>293T was validated by STR test (ATCC).<br>N-TERT immortalized keratinocytes were not authenticated.
Mycoplasma contamination	Sf21 cell line was not tested for mycoplasma contamination.<br>Human cells are routinely tested for mycoplasma contamination (average once every two months).<br>All human cell lines were tested for mycoplasma contaminated every two months using MycoGuard™ Mycoplasma PCR Detection Kit (Genecopoeia)
Commonly misidentified lines<br>(See ICLAC register)	No commonly misidentified cell lines are used in this study.
